# Applications of Machine Learning (ML) and Mathematical Modeling (MM) in Healthcare with Special Focus on Cancer Prognosis and Anticancer Therapy: Current Status and Challenges

**DOI:** 10.3390/pharmaceutics16020260

**Published:** 2024-02-09

**Authors:** Jasmin Hassan, Safiya Mohammed Saeed, Lipika Deka, Md Jasim Uddin, Diganta B. Das

**Affiliations:** 1 Drug Delivery & Therapeutics Lab, Dhaka 1212, Bangladesh; jasmin.hasan10@gmail.com (J.H.); safiya.sd7@gmail.com (S.M.S.); 2 Faculty of Computing, Engineering and Media, De Montfort University, Leicester LE1 9BH, UK; lipika.deka@dmu.ac.uk; 3 Department of Pharmaceutical Technology, Faculty of Pharmacy, Universiti Malaya, Kuala Lumpur 50603, Malaysia; 4 Department of Chemical Engineering, Loughborough University, Loughborough LE11 3TU, UK

**Keywords:** machine learning, mathematical modeling, cancer, computational oncology, tumor, telemedicine, carcinoma

## Abstract

The use of data-driven high-throughput analytical techniques, which has given rise to computational oncology, is undisputed. The widespread use of machine learning (ML) and mathematical modeling (MM)-based techniques is widely acknowledged. These two approaches have fueled the advancement in cancer research and eventually led to the uptake of telemedicine in cancer care. For diagnostic, prognostic, and treatment purposes concerning different types of cancer research, vast databases of varied information with manifold dimensions are required, and indeed, all this information can only be managed by an automated system developed utilizing ML and MM. In addition, MM is being used to probe the relationship between the pharmacokinetics and pharmacodynamics (PK/PD interactions) of anti-cancer substances to improve cancer treatment, and also to refine the quality of existing treatment models by being incorporated at all steps of research and development related to cancer and in routine patient care. This review will serve as a consolidation of the advancement and benefits of ML and MM techniques with a special focus on the area of cancer prognosis and anticancer therapy, leading to the identification of challenges (data quantity, ethical consideration, and data privacy) which are yet to be fully addressed in current studies.

## 1. Introduction

**Figure 1:**
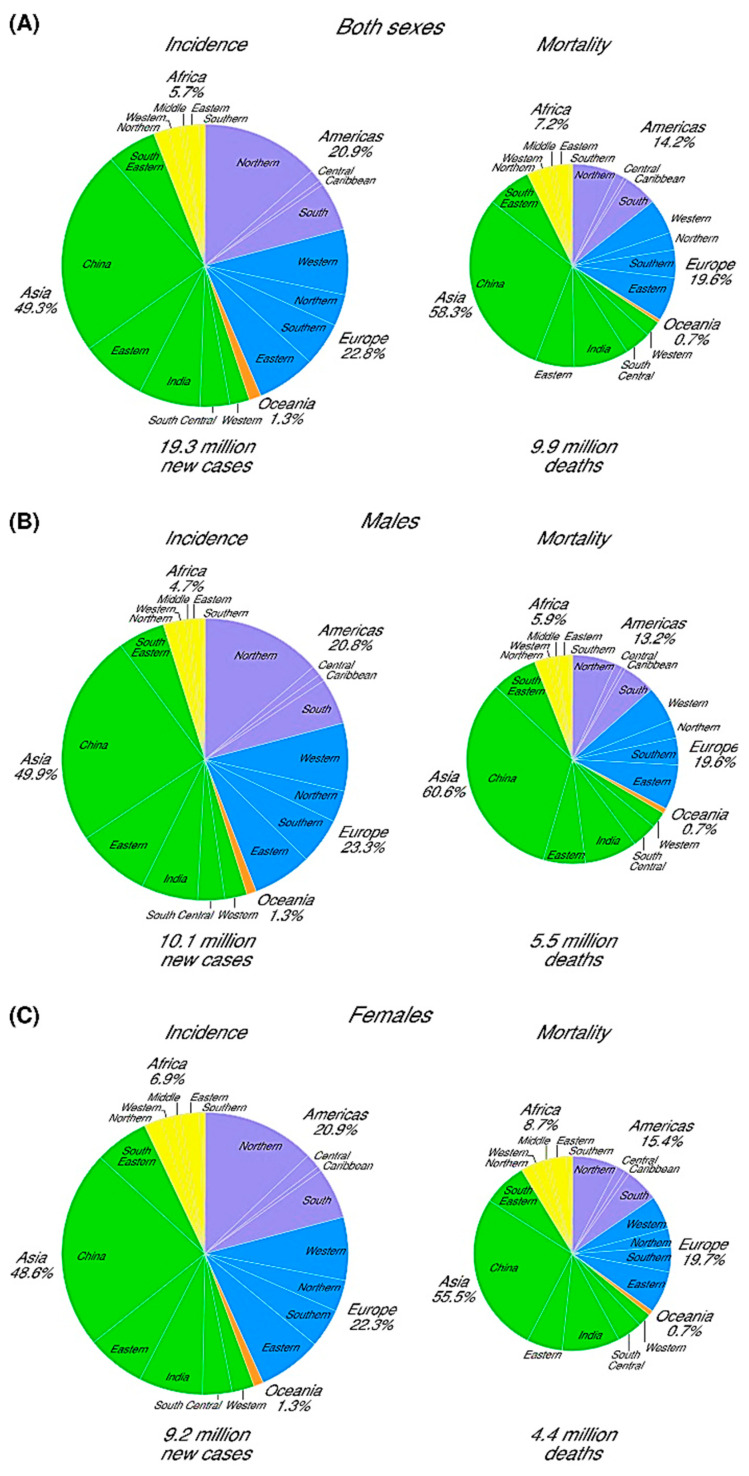
Percentage of newly diagnosed cases and deaths by areas worldwide in 2020. (**A**) Both male and female, (**B**) male, and (**C**) female [5].

Over the past several centuries, humans have made remarkable strides in the treatment of different lethal diseases; however, cancer management is still a terrifying challenge worldwide due to the aftermath in both the physical and mental health of the individuals involved [1,2,3]. There were an estimated 18.1 million newly diagnosed cancer patients worldwide in 2018, and nearly 9.6 million people died of cancer in that year [4]. The numbers for newly diagnosed patients and total deaths increased to 19.3 million and 10 million, respectively, in 2020 [5,6]. The diversity in types of cancer is the reason behind this growing rate of newly diagnosed cases and deaths each year, which leads scientists worldwide to adopt varied approaches to cancer treatment [2,7]. Figure 1 shows more detailed information regarding the newly diagnosed cases and death percentages among men and women, separately, in different geographical areas worldwide.

Currently, immunotherapy, radiotherapy, chemotherapy, targeted therapy, and surgery are the main first-line treatments for cancer [8,9]. Among them, radiotherapy, chemotherapy, and surgery fail to treat in cases of metastatic and last-stage tumors [10]. Moreover, immunotherapy has shown very restricted clinical outcomes due to factors such as the heterogeneity of cancer cells, inadequate antigen presentation, and immune checkpoint upregulation, etc. [11,12]. All of these drawbacks make it imperative to come up with a different approach that will save time and improve the effectiveness of the treatment process. Furthermore, when it comes to cancer diagnostic techniques, the visual examination and manual interpretation of biomedical images used to be typically performed before; however, these diagnostic techniques require extended time and are highly susceptible to errors. On top of that, early cancer detection is essential for saving an individual’s life. Considering these points, researchers in the early 1980s started to adopt computer-aided approaches for cancer diagnosis, prognosis, and treatment [13,14,15,16,17].

**Figure 2:**
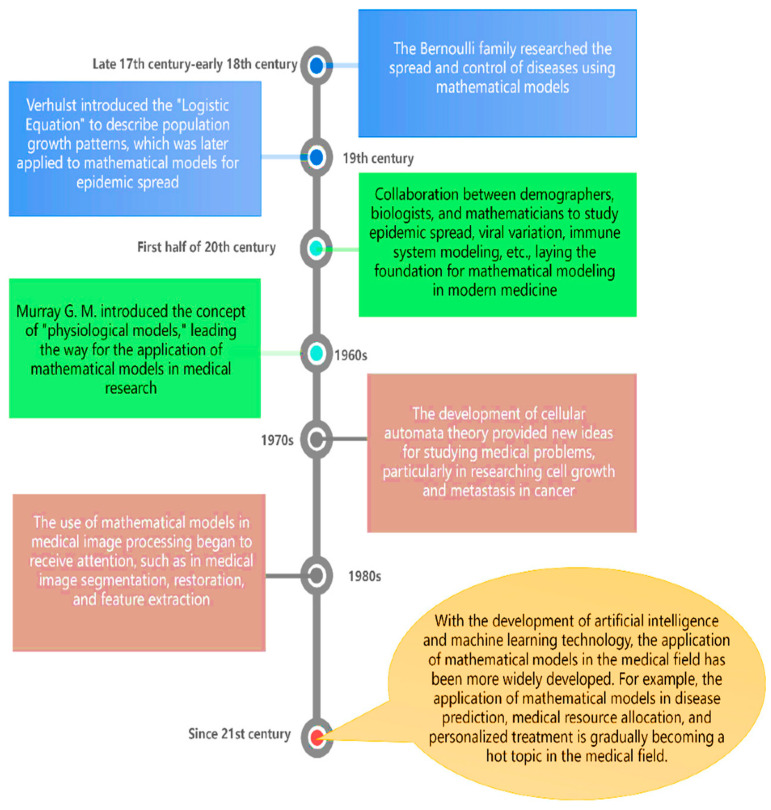
History behind the development of MMs in the field of biomedical studies [22].

As per the documentation found, it can be observed that the conceptualization of machine learning (ML) algorithms was observed around the year 1958 [18]. Since then, ML has been revolutionized substantially from only a subject of curiosity in the research laboratory to an empirical technology that is being used worldwide for practical purposes these days [19]. However, the use of mathematical modeling (MM) in biological sciences is far older than the advent of ML. This technique was first applied by Daniel Bernoulli, who formulated MM to evaluate the effectiveness of the variolation of healthy people with the smallpox virus in 1760 [20]. After that, thousands of articles that use MM have been published, and approximately 97% have been published since 1990 [21]. Since biomedical problems can be explained through MM to evaluate and predict future medical conditions, this method has gradually gained popularity because of its potential [22]. Figure 2 describes the history behind the evolution of MMs in the study of biomedical problems.

The history behind the association of ML with the field of biomedical science is long and intricate [23]. Currently, ML has created a broad wave throughout the healthcare system by assisting physicians worldwide in making quicker and more accurate clinical decisions [24,25,26,27]. Continuous technological evolution in Artificial Intelligence (AI) and ML has led the direction towards self-determining disease diagnosis tools by using big datasets to measure the future obstacles in detecting human diseases at a primal stage, especially in cancer [28,29].

Some studies show that sometimes patients are not bothered about specific diagnostic information. Rather, they are more interested in discussing prognosis, the time duration of their diseased state, the side effects of treatment, life span, the impact of cancer on their daily life, and the possibility of a cure, alongside financial liability. This puts pressure on physicians to make more accurate decisions regarding treatment and cure [30,31,32,33,34]. In addition, biomedical researchers are in search of a technological tool that can find and detect different patterns in a complex dataset and also uncover the relationships among them, and, at the same time, efficiently prognosticate the treatment outcome of a particular type of cancer, which has made them happily accept ML technology in cancer research [15].

### 1.1. General Concepts of Machine Learning (ML) and Mathematical Modeling (MM)

#### 1.1.1. ML 

ML has transpired as a subset of the artificial intelligence (AI) discipline as the method of choice for establishing empirical computer software for the purpose of natural language processing, visual and speech recognition, robot control, and many other applications [19,28]. In a broader sense, ML refers to the category of algorithms/models which help to develop tools and methods for pattern identification within datasets. The identified patterns can be used afterwards: (i) as an aid to enhance the existing knowledge regarding the current situation of the world, for example—in the biomedical field, for the risk factor identification of an infectious diseases, and (ii) predicting the future, for example—predicting the possibility of getting infected for an individual [23,35,36]. In short, the task of ML is to utilize algorithms to parse existing real-world datasets, assimilate the knowledge as an ML model, and subsequently utilize the developed model in conjunction with new data to perform the task intended, which may be classification or forecasting, etc. Some popular ML methods are decision trees (DT), the support-vector machines (SVM) algorithm, the naïve bayes (NB) algorithm, the k-nearest neighbor (KNN) algorithm, the random forest algorithm (RF), and neural networks (NN), etc. A DT is a visual representation that shows decisions and their outcomes in the shape of a tree. The graph’s edges reflect the decision, whereas the nodes indicate an event or option, regulations, or requirements [37]. Using an NB classifier, we suppose that the presence of a particular feature in a class is independent of any additional feature. NB focuses mostly on the text categorization sector. The principal applications are clustering, and the conditional probability determines the classification’s purpose of occurrence. SVMs in ML are supervised learning models with related learning algorithms that examine data used for regression and classification analyses. SVMs may effectively perform non-linear classification in addition to linear classification by implicitly translating their inputs into high-dimensional feature spaces. This technique is known as the kernel trick. In essence, it draws lines between the classes. The margins are designed to have the longest possible distance between them and the classes, which minimizes classification error. KNN is a supervised ML technique that can be used to tackle classification and regression issues. It is simple to use and comprehend, but it has the critical problem of becoming noticeably slower as the amount of data in use increases [38].

**Figure 3:**
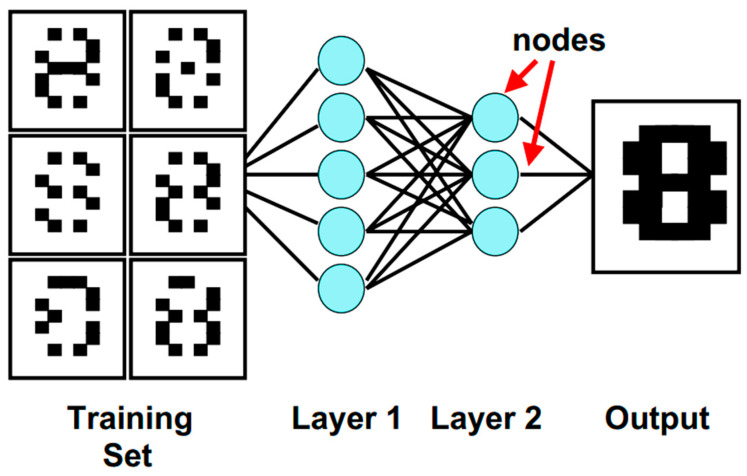
Simplified illustration for working objective of NN method of ML [16].

It is said that the ML process comprises no less than 80% of data processing along with cleaning and 20% of application of algorithms. Hence, the predictive accuracy of any ML approach depends upon the availability of a notable amount of high-quality data. [39,40]. Furthermore, to obtain an accurate outcome, an ML-based tool is required to be trained by all possible types of data generated from diverse clinical activities (e.g., diagnostic images, test results, chemical screening, identification of diseases, diagnostic errors, treatment, after treatment outcome, and unwanted events, etc.) [24,26,41,42,43,44]. Figure 3 describes the basic working objective of one of the popular ML methods, the NN algorithm.

Neural network (NN) algorithms [28], one of the most popular ML approaches, mimics the human brain in order to come up with an end result. Deep learning (DL) is another subfield of ML which is considered to be an advanced and sophisticated form of NN suitable for the identification of objects and images, the processing of languages, improvement in disease diagnosis, drug discovery, and precision medicines, and aiding humans in making clinical decisions. Another significance of DL is that, from its prior experiences, it can propose an output as well [28,45,46]. Moreover, with an artificial neural network (ANN), it can also analyze data that contain medical images mimicking the neuronal architecture of humans and consists of an input, output, and variety of hidden multi-layer networks to improve the ML processing ability [47,48].

Real-world data are mostly inconsistent. Since AI algorithms generally deal with large datasets in the biomedical field, it becomes essential to shape these data into being submission worthy through data pre-processing (DP), so that it can be inputted into the desired ML algorithm for further processing and predictive results [49,50]. The DP procedure involves three segments, which are: (i) data reduction, (ii) data projection, and (iii) missing data treatment, and these segments involve multiple methods as well [51]. To understand these methods, we also need to understand the following terms along with them. 

A *dataset* is basically a set of information that is provided to train the ML tool for predicting future events [52]. It forms the foundation for training and analyzing ML models. Also, it is engaged in the fundamental role of further developing the process. Moreover, it gives information regarding the issues of a particular field and the approach methods to develop algorithms out of the process of data collection, construction, and allocation [53].

*Principal component analysis* (PCA) holds the most importance, being a classical tool for the analysis of datasets. It can be implemented on a given raw dataset without any prior training. This trait of PCA enables computers with lower specifications to perform better [54]. In PCA, the equation for a measured matrix is $M\in {\mathbb{R}}^{\left(n_{1}\times n_{2}\right)}$. Here, *n*_1_ signifies the number of sample dataset and *n*_2_ signifies the number of variables. PCA has two more variants, such as robust principal component analysis (RPCA) and kernel principal component analysis (KPCA) [55]. Recently, Kang et al. proposed one more PCA method called self-paced principal component analysis (SPCA) [56]. These PCA methods are discussed briefly in this paper for the completeness of the discussion. 

**RPCA:** This is the refined version of PCA by the decomposition of a measured matrix [57,58]. For instance, if a measured matrix is *M*,

(1) $$M\in {\mathbb{R}}^{\left(n_{1}\times n_{2}\right)}$$

RPCA decomposes Equation (1) into a lower-grade matrix *L* and a sparse matrix *S*. Hence,

(2) $$L\in {\mathbb{R}}^{\left(n_{1}\times n_{2}\right)}$$

(3) $$S\in {\mathbb{R}}^{\left(n_{1}\times n_{2}\right)}$$

By decoding the principal component pursuit (convex program) [55,59]:

(4) $$min_{L,S\;}\|L{\|}_{*}+\lambda \|S{\|}_{1\;\;\mathrm{subject}\;\mathrm{to}}M=L+S$$

Here, ‖⋅‖_∗_ = norm of the matrix 

‖⋅‖_1_ = *L*_1_-norm of a matrix 

and *λ *= tuning parameter, as interpreted in [55,58].

We can evaluate the value of *λ* using the following equation:

(5) $$\lambda =\frac{1}{\sqrt{max\left(n_{1},n_{2}\right)}}$$

From the estimated value, we can further fine tune it [55].

**KPCA:** This is the refined version of PCA as well. Using a kernel function, it maps raw data into a new (feature) space *F* and, subsequently, the classical PCA algorithm is applied in *F* [60,61]. Typically, PCA performs well when it is linear variation of the data, whereas its performance is bad with non-linear variation of the data. As per the Cover’s theorem, a non-linear dataset sample obtains a linear form after it is mapped to the feature space *F*, which can be delineated by a kernel function. KPCA can handle both linear and non-linear data [62]. 

The data collection process is costly. Moreover, due to the carelessness of the associated teams, missing data seem to appear quite frequently in a dataset [63,64,65]. *Missing data* have a notable impact on the performance of ML algorithms [66]. On account of this, it is required to implement the missing data treatment (MDT), which involves the deletion of missing data or replacement with their estimates [63,67,68,69]. The MDT can be executed by applying two strategies, such as (a) the deletion method and b. imputation methods. The deletion method has two types, which are (i) listwise deletion and (ii) pairwise deletion, and the imputation method includes many types such as regression imputation, mean imputation, hot-deck imputation, and cold-deck imputation, etc. [51,64,69,70,71]. 

*Scaling* is a vital step for ML techniques, which refers to the transformation of feature data, as per defined commands, to the same scaled data that have similar level of impact. Therefore, the technique is correctly encrypted to the selected units [51,72]. Generally, two intervals are used as scaling targets, which are [0, 1] and [−1, 1], which are interpreted as follows,

(6) $$\left[0,1\right]\;\mathrm{interval}=\frac{\mathrm{actual}\;\mathrm{Value}\;-\;\min\left(\mathrm{all}\;\mathrm{Values}\right)}{\max\left(\mathrm{all}\;\mathrm{Values}\right)-\min\left(\mathrm{all}\;\mathrm{Values}\right)\;}$$

(7) $$\left[-1,1\right]\;\mathrm{interval}=\frac{\mathrm{actual}\;\mathrm{Value}\;-(\max\left(\mathrm{all}\;\mathrm{Values}\right)+\;\min\left(\mathrm{all}\;\mathrm{Values}\right))/2}{\left(\max\left(\mathrm{all}\;\mathrm{Values}\right)-\;\min\left(\mathrm{all}\;\mathrm{Values}\right)\right)/2}$$

It was observed from a survey conducted by Huang et al. that the [0, 1] interval was considered to be used for scaling by default in most of the studies [51]. 

Despite the fact that ML is useful for intensive data analysis, this study shows that the use of high-dimensional data, also known as multivariate data, for training and analyzing decreases its reliability and performance. Multivariate data are common in datasets and make it hard to contrive information from the analyzed data for ML algorithms [73,74,75]. Trying to solve the complex problems of the real world using these multivariate data without an increase in sample size causes *blind spots* (continuous sections of feature space without any scrutiny), which creates challenges while developing the model. This lack of ability of ML to manage multivariate data is familiar, as the *curse of dimensionality* is one of the major drawbacks of ML [76,77]. For instance, the Watson supercomputer was trained using a small sample size of 106 ovarian cancer cases and 635 lung cancer cases. A small multivariate data sample would be highly inclined to cause a *blind spot* here. Now, if data from this blind spot come across after deployment, an inappropriate treatment recommendation can be produced, which is not detected while developing the model [76].

**Figure 4:**
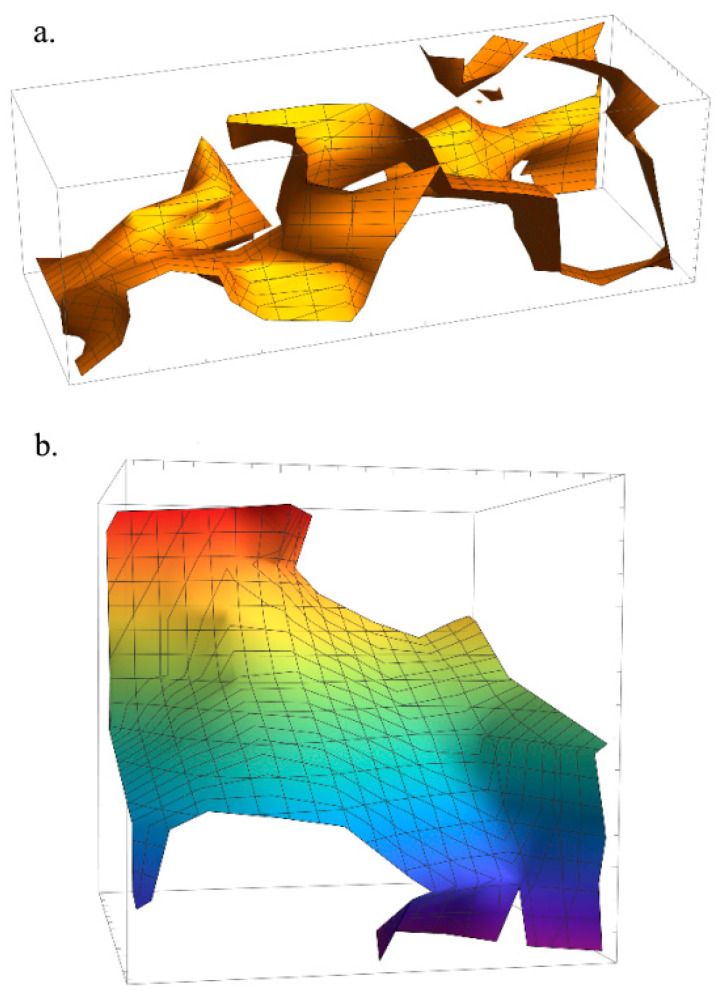
An example of the curse of dimensionality. (**a**). Outcome of multiple discontinuities (**b**). Outcome of continuous sensor computation [77].

A dataset contains data points (objective) which are described through a number of features (variables, where the number of variables is fixed). These features can be of two types: (a) continuous (continuous numerical values) and (b) categorical (only discrete values). Mostly, this categorical feature is merely binary and functions by true or false, in binary, 1 or 0, respectively [78]. Raw data can act discontinuously during the computation of continuous sensors (see Figure 4) because of the diverse range of parameter variations and high dimensionality because of multi-sensor computation. 

*Dimensionality reduction* is necessary to avoid the misestimation of original data via ML algorithms. Datasets must be pre-processed for the prognosis of any human disease. At this point, dimensionality reduction has an important role in multivariate data’s dimension reduction. It requires the mapping of higher-dimensional inputs into lower dimensions so that almost identical points in the input space are mapped into neighboring points on the manifold learning, which is the procedure of non-linear dimensionality reduction, and another is linear dimensionality reduction [79,80]. Apart from that, there are two ways to reduce dimensionality, which are, (a) *feature selection* and (b) *feature extraction*. 

Mathematically, for instance, there is an *n*-dimensional vector denoted as *X.*

*X* = [*x*_1_, *x*_2_, …. *X_n_*]^T^ (8)

Now, *X* is mapped to an *m*-dimensional vector denoted as Y through a map *f*, where,

*Y* = [*y*_1_, *y*_2_, …. *Y*_m_]^T^ (9)

and, *m* < < *n* (10)

The condition is m-dimensional vector Y, which should contain the principal features of *n*-dimensional vector *X*. 

So, the mapping function can be expressed as:

*Y* = *f* (*X*) (11)

This is the fundamental mathematical process of feature selection (FS) and feature extraction (FE) [81,82]. Here, mapping *f* is the algorithm that needs to be found for feature reduction, and the choice differs depending on the pending real-world problems [83].

FS is a process of feature subset selection that is implemented on the construction of a model [84]. FS differs from dimensionality reduction, as FS does not change the original features but simply selects a subset, whereas dimensionality reduction could comprise creating new features to preserve all features. The prior condition of using FS is to delete the redundant features of data without losing the necessary information. The purposes of using FS are, (a) model simplification to make it user-friendly to interpret, (b) cutback on run time, (c) subside curses of dimensionality, and (d) variance reduction [83]. On the other hand, FE produces (map) new features of data from the original ones. Its benefit is the efficient compression of the mapped features, and its drawback is the mapped feature set might lose meaning, although the original one has a clear structural meaning. FE executes two functions that are (i) necessary detail separation from redundant data, and (ii) classifier performance reduction via decreasing the dimension [85,86]. Some commonly used FS/FE techniques are PCA, linear discernment analysis (LDA), discrete Fourier transform (DFT), factor analysis (FA), independent component analysis (ICA), and an autoencoder [79,81,84,87]. 

**Figure 5:**
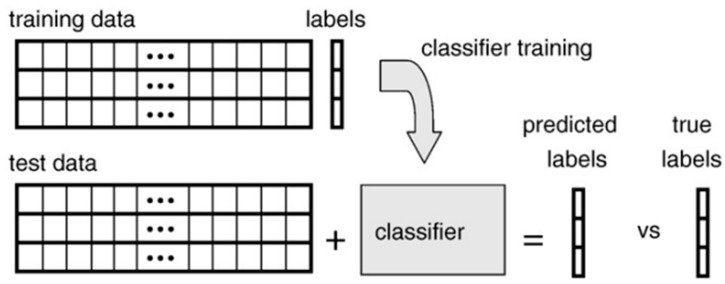
General working process of a classifiers [88].

*Classification* is the activity of predicting the dependent variables by analyzing the parameters and values of different features in a set of independent variables (Figure 5). A variety of parameters is learned by a classifier from a training dataset. A distinct dataset is returned by a classifier afterwards. If the values of this dataset can be made to be mutually exclusive, in that case, they are termed as *class*, and they do not need to be mutually exclusive, in that case, they are called *label*. As an example, the residue of a protein is supposed to be in only one of the multiple secondary structure classes. However, at the same time, it could be assigned to the non-exclusive labels of being transmembrane and ⍺-helical. They are generally denoted by an encoding. A trained classifier is basically a representation of the interconnection between the voxels (raw/test data) and the class label from a training dataset. Mathematically, if the voxel denoted as *x* and classifier denoted as function f that predicts the class label denoted as y, then this can be demonstrated as: *y* = *f*(*x*) [78,88,89]. There are a variety of classifiers in ML that are used in cancer research which will be mentioned later on. 

From the aforementioned sequence of discussion regarding the techniques or terms used in the ML algorithms throughout Section 1.1.1, we can obtain an idea of their working processes.

#### 1.1.2. MM 

**Figure 6:**
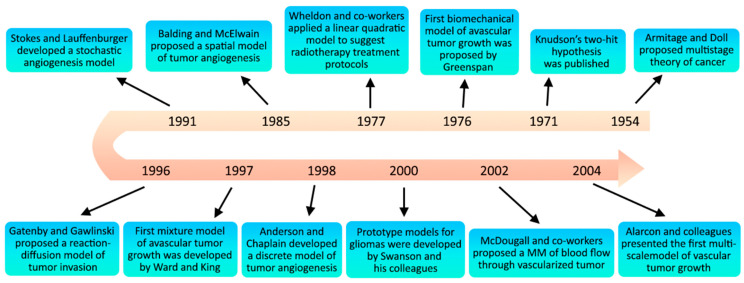
History of MM in the field of cancer research.

MM can be defined as the art of representing real-world issues through mathematical terms to predict a probable future or provide an insight [90]. It is customarily a schematization of the real-world situation and, therefore, based on the objectives and the available variables, it is possible to develop different MMs for the same incident [91,92]. MM is not new in the field of cancer research (Figure 6). There are many hypotheses with respect to cancer which are required to be tested in the laboratory and run in in vivo experiments. However, this is time-consuming, costly, and sometimes not possible, also due to the lack of appropriate technology or the potential involvement of humans. In such conditions, MM plays an alternate role in checking the potential of these hypotheses. If the hypotheses fail to exhibit the claims, then they must be revised before proceeding further towards the lab [93,94].

One of the initial MMs in cancer research is that of Armitage and Doll’s multistage theory, which explains the series of mutations a cell undergoes before becoming cancerous. It also mentions that the risk of cancer development grows with the power of a person’s age. Supposing the power of age is five, then a cell has to go through four stages to become cancerous [95,96,97,98]. The mathematical illustration of this model is as follows [99]:

1.

(12) $$I=\frac{N_{p_{1,}p_{2,}p_{3,}p_{4,}\ldots \ldots \ldots p_{r}}}{r-1!}\;t^{r-1}$$

2.

(13) $$I=N_{p_{1}}\left(1-e^{p\frac{2}{k}\left(e^{kt}-1\right)}\right)$$

Here, *k* = a constant

*N* = Number of cells at risk

*I* = Cancer incidence

*p* = Probability of change in any cell at any age 

*r* = Number of changes [99].

Even though this theory provides an outstanding demonstration of the cancer incidence rate in terms of stomach, pancreas, and colon cancer, it is unfit to demonstrate others, which include prostate and breast cancer. Moreover, it does not present any mechanistic insight regarding the bio-functional changes accountable for the progression of cancer [100,101]. This Armitage and Doll’s theory was motivated by the study of the mortality statistics of cancer, which was proposed by Nordling in 1953 [98]. 

Discussing historical models, the linear-quadratic model is one of the classical MMs in radio biology, which gives us insight into the relationship between cell survival probability and a single dose of radiation. Around 50 years have passed since this model was first proposed, and eventually, it has become the method of choice for both researchers and clinicians for characterizing the effects of radiation on cells [102]. It can be represented mathematically as follows:

(14) $$S=e^{-\alpha D-\beta D^{2}}$$

Here, *S* = A single dose of radiation

*α* = Linear parameter for radio-sensitivity of a cell at a low dose

*β* = Quadric parameter for radio-sensitivity of a cell at a high dose

*D* = Dose at which cells are vulnerable [102].

The same as Armitage and Doll’s theory, this linear-quadratic model also has limitations. In an article published in 2020, Loap and co-workers mentioned the linear-quadratic model as being questionable at the tumor level, since this model talks about the probability of surviving a single dose of radiation therapy for a given single cell type. This is a fact, because when it comes to tumor cells, the real scenario is far more complex than we can even think, due to the complexity of the micro-environment a single cell carries [103].

These early models seem quite simple compared to the MM models being developed to analyze solid tumor growth at present [104,105]. We will discuss the newly formulated approaches with their applications in the study of cancer prognosis and treatment later. 

In this review article, we will discuss both the ML and MM approaches recently undertaken to improve cancer prognosis and treatment procedures and their related challenges.

## 2. Paradigms of ML

**Figure 7:**
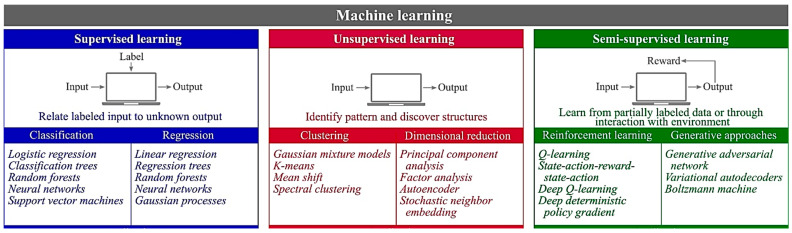
Classifications of ML [108].

Supervised and unsupervised approaches are the two main paradigms of ML [15,23]. In supervised learning, a trained dataset is applied to labeled data with the purpose of formulating a system that is capable of accurately predicting the type of raw dataset based on available features. It is also applied to predict the categorical (class/label) characteristic, which is known as discriminant analysis, or the continuous characteristic, which is known as regression analysis. Contrarily, in supervised learning, no class/labeled data are provided while mapping the algorithm. Hence, it is capable of predicting the patterns of non-labeled data without being trained by a predefined class/label dataset [15,23,106,107]. Another type of ML is semi-supervised learning, which is the intersection of supervised and unsupervised learning and learns from both the labeled and unlabeled types of data [108]. Figure 7 provides an overview of these three ML categories with their further classification.

### 2.1. Supervised ML

In supervised learning, the classifier returns a value of doubt or an outlier. Doubt indicates when the decision making is unclear. For example, it is unsure of the new data and which class/label they would be fitted to assign. An outlier indicates the unlikeliness of new data compared to any of the previously observed data, which makes their possibility to be predicted as something certain questionable [23].

Classification supervised learning identifies certain entities and examines them to determine how to categorize them when labeled, whereas regression is also a kind of supervised learning that gains knowledge from labeled datasets to forecast continuous results for various inputs in an algorithm. To comprehend the connection between reliable and independent variables, regression is employed. It is frequently employed in situations where the output must be a finite value, such as when determining a person’s height or weight, etc. Further subcategories include: 

For classification: (i) DT; (ii) SVM; (iii) NB; (iv) NN; (v) KNN; (vi) RF; and (vii) linear classifiers; and for regression: (i) simple linear; (ii) multiple linear; (iii) polynomial; (iv) SVM regression; and (v) logistic [109]. Supervised ML learning in biological and healthcare innovations demands knowledge-based health management systems, quality data consciousness, and expertise. There is a known workflow for supervised ML learning methods in the field of healthcare, established by Roy and co-workers. They illustrated the following workflow: (a) data collection (structured/non-structured data); (b) data processing (implement, foresee, understand, and take appropriate action); (c) outcome (achievement rate and its emerging issues); (d) evaluation (analysis, execution, and its subsequent response); and (e) validation (thorough correction and verification in light of further data) [110].

### 2.2. Unsupervised ML

In unsupervised learning, the purpose is to inspect the data and identify similarities among them. These similarities characterize a group of data, which is referred to as a cluster. To be precise, it is used for the revelation of naturally occurring groupings in the data. So, in this case, no data are labeled, and the learning procedure comprises characterizing the nonlabeled data by matching the raw data with them [23,111].

For evaluating high-dimensional data, such as those from transcriptomic, metabolomic, and proteomic research, clustering is frequently utilized. The key influences on the readouts and modules with a high degree of coregulation are often identified using hierarchical clustering. Non-hierarchical clustering is used in single-cell sequencing to recognize the different cell types present in the sample. In order to find associations between individuals, tissues, illnesses, or even disease symptoms, clustering is also employed. In order to direct drug discovery, drug compounds may also be grouped based on the characteristics of target proteins, such as sensitivity and gene expression [112,113,114,115,116]. In transcriptomic and other -omics investigations, dimensionality reduction is frequently utilized to find outliers and possible batch implications. Dimensionality reduction may also be employed as a pre-processing stage to improve the algorithmic efficiency of an ML model or to comprehend the high-dimensional chemical space [117,118]. 

### 2.3. Reinforced Learning

Reinforcement learning is a semi-supervised ML technique that focuses on engaging with its surroundings by taking action, learning from its mistakes, and identifying patterns. It tries several behaviors to see the ones that maximize the cumulative reward in an environment instead of being given instructions on what to do, whereas supervised ML employs a collection of input and output data pairs for training. Q-learning, hierarchical reinforcement learning algorithms, temporal difference learning, and policy gradient algorithms are a few examples of well-known reinforcement learning algorithms. The summary of long texts is one real-world business use of reinforcement learning [111,119].

Although reinforcement learning has been around for a while, its applications in healthcare are just beginning to shine. Recently, a sensor-assisted pump was shown to be outperformed by a closed-loop control system created by combining an ML-type control algorithm with structural PK/PD models that are already in use and are well-known to pharmacometricians [120]. Another study by Popova et al. [121] focused on using a variety of ML methods to combine novel molecules via traditional reinforcement learning, of which 95% of the molecules obtained were practical [121]. One study employed reinforcement learning to tailor anemia therapy via pharmacological means [122]. Reinforcement learning was employed by Turki and Taguchi [123] to speed up the process of finding potentially helpful medications. They showed that the process of identifying drugs lasted only 46 days, which is a significant reduction from the time required by traditional approaches. They concluded that various algorithm coding changes still need to be performed to assure that synthesized chemicals have distinct formulae from items currently on the market [123].

## 3. ML and MM Approaches in Healthcare

The purpose of ML and MM is to create predictive outcomes of a certain phenomenon [124]. ML utilizes data and algorithms shaped by using MMs to simulate how people learn. Over time, as more good data are submitted to ML approaches, their accuracy also increases. By utilizing data collected from patients and minimizing human engagement in analysis, ML is utilized in healthcare to enhance the efficacy and overall quality of treatment. A useful feature of ML is that, once it understands the method to solve a given problem, it provides the solution at a faster rate, as its algorithm has the information implanted, with zero chances of error. These features strengthen the base for ML to facilitate healthcare services and may assist in varied clinical capabilities, from giving medical assistance to the overall automatization of the entire clinical system for the ease of task management, in order to minimize human intervention [125,126,127].

ML and MM can operate healthcare management and services with full efficacy, as there is a specific goal, and its utilization will provide the quickest but most efficient way to come up with a solution, thus reducing human time and effort for menial tasks. By removing the human’s role from the analyzing system, these technologies can decrease mistakes and execute repeated tasks with more efficiency than manual efforts [128,129]. 

The same ML approach can be used to solve varied problems. According to Goecks et al., in a clinical context, ML will be used to analyze high-fidelity imaging and molecular tests so that medical practitioners can find notable biomarkers to obtain a final diagnosis [130]. Multiscale modeling and automated search results for comparable patient conditions will be used to help make treatment decisions for an unknown disease [131]. Following diagnosis and treatment, health management recommences with continuing personal health monitoring, and for that, an ML system must achieve many objectives, which includes tracking the patient’s response to therapy, keeping an eye out for potential negative effects, tracking general health, and tracking the changes from the starting point that are unrelated to the course of treatment [130,132,133].

### 3.1. Discovery of New Drug Molecule

**Figure 8:**
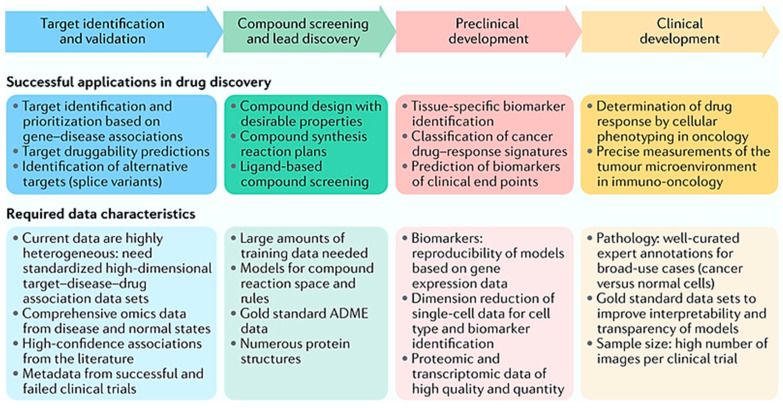
Applications of ML and MM in new drug discovery [39].

Introducing a new drug molecule to the pharma market is a vast area of research in the field of both biomedical and pharmaceutical sciences. There are obstacles in the way of drug discovery and development, for example, high expenditure, consumption of time, off-target delivery, lower efficiency, complex omics data, and lengthy clinical trial phases. Nowadays, new drug molecules are being introduced to the market due to the successful applications of ML and MM in varied phases of drug discovery and development by researchers (see Figure 8). The advancement of ML algorithms makes the whole process rationale cost effective and, all in all, more beneficial to humankind [134].

Target identification and priority setting are the first steps in the conventional target discovery process. This involves the discovery of a target with a causal relationship with some component of a pathophysiology and a convincing argument for the idea that modulating this target will modulate the illness itself [135]. Target identification is, without a doubt, an important step along this road, even if evidence of a successful treatment approach will initially come from in vivo drug response studies followed by demonstrating efficacy in a randomized clinical trial [136]. The discovery and verification of chemically active substances, target detection, protein production, the assessment of medicinal contaminants and physicochemical characteristics, medicinal surveillance, the assessment of medicinal effectiveness and efficacy, and drug reposition are all made possible by computational modeling built around ML principles [137].

For well-defined problems with a lot of useful data, ML techniques offer a set of tools that can enhance discovery and decision-making processes. ML applications are possible at every level of the drug development process. Examples include the discovery of prognostic biomarkers, target validation, and the evaluation of digital pathology data in drug trials. The creation and implementation of ML algorithms and software have begun at all stages of drug discovery and development, such as clinical trials, the identification of novel targets, strengthening proof for target–disease interactions, enhancing small-molecule compound design and optimization, increasing understanding of disease mechanisms, raising understanding of disease and non-disease phenotypes, constructing new biomarkers for prognosis, progression, and drug efficacy, and improving the analytical methods for biometric and other data from patient monitoring and wearable devices, improving digital pathology imaging as well as extracting high-content information from images at all levels of resolution [138,139,140,141,142,143]. Table 1 describes the features of ML tools used in drug discovery and design.

### 3.2. Prediction and Management of Global Pandemic

Pandemic management is way more costly than the cost of avoiding them. For instance, the global cost of the severe acute respiratory syndrome (SARS) outbreak was an estimation from USD 13 billion to 50 billion for the 2003′s single outbreak [150,151]. The use of MM and ML can aid at this point and, for this reason, in the recent COVID-19 pandemic, researchers applied varied approaches using ML and MM for prediction and management to minimize costs [152,153,154,155,156,157].

Several papers on accurate and early identification related to COVID-19 using ML along with MM have been published. Accumulated case reports, medical pictures, management techniques, personnel in the healthcare industry, demographics, and migration during the outbreak helped in preparing some of the datasets that are now available. ML and MM demonstrated themselves to be effective tools in the battle against this epidemic. Additionally, a number of COVID-19-related datasets have been gathered and made available as open source. Both ML and MM have exhibited some working models for the prediction and management of the COVID-19 pandemic. In the literature, several research projects were produced for the simulation of COVID-19 behavior and dissemination. For MM, the Susceptible–Exposed–Infected–Removed (SEIR) model and the Susceptible–Infected–Recovered (SIR) model were the main models upon which the majority of these were built. In the past, these models were heavily utilized to explore how epidemics spread across different types of transmission networks [158,159,160,161]. For ML, Naive Bayes (NB), Convolutional Neural Networks (CNN), Linear Discriminant Analysis (LDA), Logistic Regression (LR), Random Forest (RF), Support Vector Machines (SVM), and Decision Trees (DT) were a few of the supervised learning algorithms used to identify COVID-19. However, there is still more to be conducted to diversify these databases [162]. Table 2 summarizes the MM models studied for the prediction and management of COVID-19, whereas Table 3 shows the different ML algorithms employed for COVID-19, along with their accuracy.

### 3.3. Epigenomics

The study of genetics has made substantial use of ML techniques (Table 4) [197,198]. According to the Encyclopaedia of DNA Elements (ENCODE), a genomic sequence which either encodes a specific product (such as a protein or noncoding RNA) or has a recurrent biochemical attribute (such as being attached to a protein or bearing a specific biochemical mark) is referred to as a functional element. The second stage of the ENCODE project, which covers the whole genome, was explained by De Souza [199]. The majority of genomics databases, most notably those produced via sequencing, are usually made available to the public for free. The majority of genomics journals need public access identification for each dataset connected to a publication, serving as proof of this practice. This widespread embrace of data openness may be a reflection of how the field of genetics has developed over time [200]. A few databases and tools have been developed to make genomic analysis feasible, such as StemBase [201], PluriNetWork [202], FunGenES [203], Plurinet [204], iScMiD [205], and SyStemmCell [206]. But, generally, all these databases have substantial amounts of information about transcriptome measurements and leave other essential information unnoticed. Thus, there are calls for further data integration, which brings us to the Embryonic Stem Cell Atlas from Pluripotency Evidence (ESCAPE). This is a database that accommodates a great deal of diverse data, ranging from transcriptomics and epigenetics to proteomics and phosphor proteomics. These datasets are organized as protein–protein and gene–gene interactions, gene lists, and data tables. Hence, they can be easily downloaded and utilized as per the user’s desire [207]. Classifications of metastatic brain tumors, prostate cancer, coronary heart disease, neurodevelopmental disorders, and tumors of the central nervous system are a few examples for the utilization of epigenetic data by ML [208,209,210].

ML algorithms are applied to epigenetic data because of their characteristics, and support vector machines (SVM), random forests (RF), LASSO regression, non-metric multidimensional scaling, logistic regression, convolutional neural network, and stacked denoising autoencoders are some examples [211]. Additionally, numerous samples are made available through large-scale, data-rich archives like The Cancer Genome Atlas (TCGA), ENCODE, and the BLUEPRINT project, so that thorough, high-throughput statistical studies can be performed [212,213]. Such repositories might offer an ML algorithm’s training data or an independent test set to assess the algorithm’s external validity and eventual clinical applicability [214,215]. The creation of these databanks is a crucial step, since ML algorithms need an extensive amount of data to generate accurate predictions. The majority of datasets are made up of DNA methylation profiles that were obtained from peripheral blood; therefore, patients just need to give a tiny quantity of blood. The utilization of peripheral blood as a gauge of DNA methylation may be less beneficial in diseases like certain malignancies, with higher clinical relevance in conditions like obesity [216]. DNA methylation patterns that are tissue-specific should also be highlighted [217,218].

**Table 4:** ML models with different epigenomic purposes along with key challenges.

Area of Purpose	ML Tools	Prediction Details	Challenges	Reference(s)
Protein sequencing	ResNets, 2D convolutional neural networks (CNNs)	Structure	Data accessibility is tough, and leakage of these data make the evaluation tougher	[219]
	Multilayer perceptrons with windowing	Function		[220]
	Transformers	Protein–protein interaction		[221]
Gene sequencing	1D CNNs	Accessibility of genome	Genome contains repetition of codes	[222]
	Recurrent neural networks (RNNs)	Arrangement of 3D genome	Missing data of interest	[223]
	Transformers	Interactions between enhancer and promoter	Lengthy sequences	[224]
Genetic expression	Clustering	Intergenic interactions or co-expression	Link between function and co-expression is not clear	[225]
	CNNs		Multidimensional	
	Autoencoders	Organizing transcription machinery	Loud noise	[226]
Interactions between proteins	GCNs	Side effects of poly-pharmacology	Networks for interactions can be incomplete	[227]
	Graph embedding	Protein function	Protein’s interaction depends on cellular location	[228]
			Number of possible combinations is higher	

### 3.4. Protein Engineering

The goal of protein engineering is to create or find proteins with features that can be applied in technical, scientific, or medical fields, whereas the basic objective of ML-guided protein engineering is to locate variants that differ from wild-type sequencing in terms of a certain feature. The amino acid sequence of a protein affects factors associated with its function, including its expression level and catalytic activity. This link is reversed in protein engineering to identify a sequence that fulfils a certain function. However, the functional levels of closely related proteins cannot be distinguished using existing biophysical prediction techniques. Furthermore, the number of potential proteins is too huge for a thorough natural laboratory or computer search [229,230,231,232].

The phases of protein sequences and the corresponding functional measurements of the proteins are used to train ML models. What the model can learn depends on the examples that were used to develop the model. It is possible to choose the initial set of variations to screen randomly from a library [233] or to gather as much information as possible about potential mutations [234,235]. The most straightforward approach is typically to choose variations at random. However, for low-throughput screens, it might be crucial to optimize the information gained from expensive tests, because doing so would increase the model accuracy for undiscovered sequences. Maximizing the variety in the training sequences is approximately similar to maximizing knowledge about the rest of the library. The user must choose the sort of ML model to employ, train the data in a suitable way, and then train the model after gathering the first training data.

Decision trees (classification and regression trees), RFs, SVMs, and Gaussian process models are implemented in protein engineering [229]. Due to the promising outcome of ML in protein engineering, new ML models and techniques have been developed quickly, necessitating the creation of strict performance standards and criteria for model comparison. Comparing ML modeling techniques is challenging, since different datasets, train-test splits, and evaluation criteria have different effects on model performance [236]. Designing clever combinatorial libraries for controlled protein evolution is also an attractive use of ML in protein engineering [237].

## 4. ML Algorithms in Specific Types of Cancer

### 4.1. Lung Cancer

Lung cancer causes the maximum number of cancer-related fatalities. Its percentage in men is 29.2% for those aged from 45 to 64 and 22.8% for those aged 65 and above, and in women, 17.9% for those aged from 45 to 64 and 13.7% for 65 and older [238]. The diagnosis of lung cancer includes chest computed tomography, bronchoscopy, sputum cytology, video-assisted thoracoscopy, and lymph node biopsy, etc. [239]. Imaging tests that are computed tomography scans (CT scans), magnetic resonance imaging (MRI scans), and positron emission tomography scans (PET scans) have specific limitations. With imaging tests like CT and PET scans, the absence of repeatability is a well-known issue in radiomics. This is mostly due to the lack of defined procedures and settings throughout the workflow [240,241]. Numerous studies also experience significant constraints at the stage of validation. Inadequate statistical evaluation (such as failing to adjust the p-value across several tests) and/or the lack of a standalone validation dataset to support the findings are two examples. This might easily result in skewed discovery rates and increase type-I mistakes [242]. Publication bias also tends to exaggerate good outcomes in contrast to negative ones [243,244]. Expense and accessibility are two obstacles that discourage the general public from implementing CT scans. However, the chance of developing lung, breast, or thyroid cancer over time is also increased by low-dose radiation exposure, particularly in patients who have a history of having several CT scans. False-positive results from low-dose CT scans can force patients to undergo more intrusive procedures like biopsies and surgery to remove the abnormality, which comes with extra intra- and post-operative risks and problems [245,246]. Even sputum analysis shows similar limitations, which are: (a) inadequate sensitivity or accuracy during analysis and (b) failure to detect small-diameter carcinomas. However, these particular limitations could be overcome by immunostaining [247]. 

**Figure 9:**
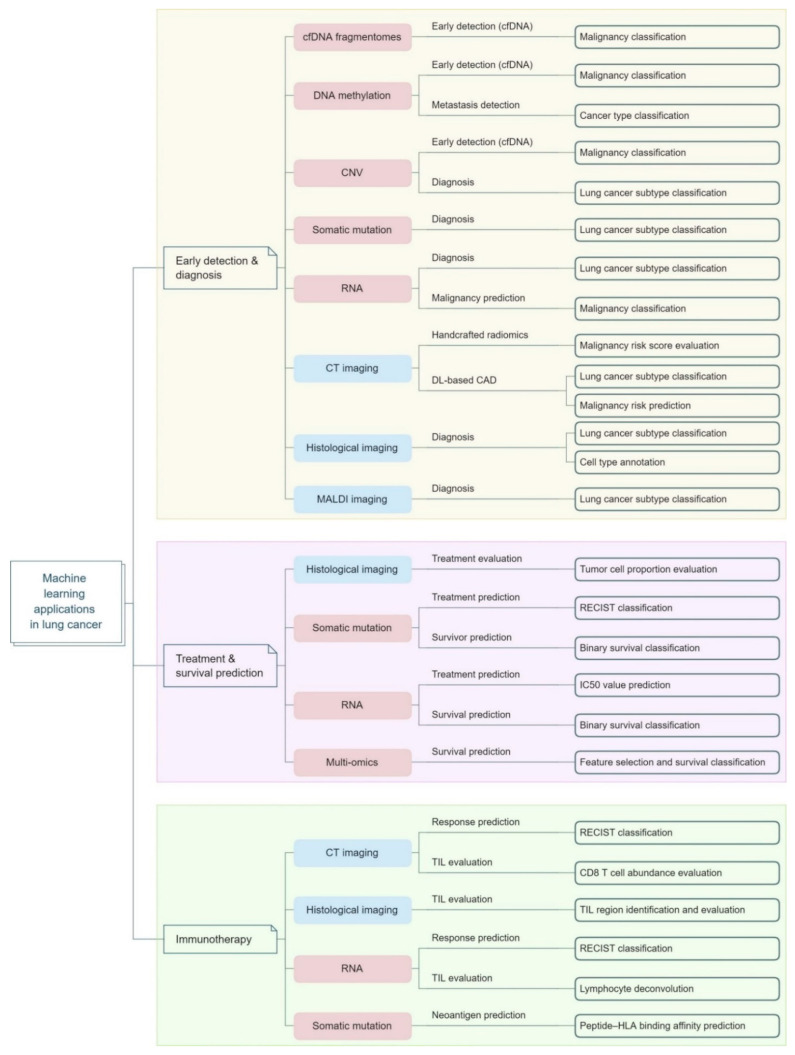
The applications of ML algorithms in lung cancer management [258].

ML helps to minimize or overcome these limitations and go above and beyond to save time and minimize errors by increasing accuracy and sensitivity to the diagnosis. For early lung cancer diagnosis, some methods use the clinical data from the patient along with the observed texture properties of specific nodules in CT images as input features to train ML classifiers, such as Logistic Regression (LR) or Linear Discriminant Analysis (LDA), for estimating the likelihood of malignancy. Clinical factors include the patient’s age, gender, specimen collection timing, family history of lung cancer, and smoking exposure, etc. Usually, these metrics include nodule size, type, location, count, border, and emphysema information in CT scans [248,249,250]. One study focused on using six different ML classifiers (Support Vector Machine (SVM), Naïve Bayes, Random Forest, K-nearest neighbor, Neural Network with 10-cross fold methodology, and AdaBoost) to determine the best lung tumor forecast depending on metabolomic biomarkers features. They gathered a total of 110 lung cancer patients, as well as 43 individuals in good health. For early lung tumor prediction, Naive Bayes is advised as a useful method [251]. Large pan-cancer sequencing datasets from earlier ML research have demonstrated the effectiveness and progress of early detection and cancer type classification, which may support lung cancer diagnosis [252,253,254]. Cancer cells exhibit a wide range of genetic abnormalities, and the aggregation of these differences can serve as markers to identify the mutational patterns of various cancer types. To improve the accuracy of ML models, recent research has focused on obtaining better genetic signatures as input features. Blood-based liquid biopsy (cell-free DNA fragments, circulating tumor DNA, microRNA, methylation, exosomes, and circulating tumor cells) is regarded as a reliable technique for early identification to examine prospective circulating tumor markers [255,256]. Even Gould et al. performed a study on AUC, sensitivity, and diagnostic odds ratio, which are used as performance indicators in developing a model to predict a future diagnosis of lung cancer based on routine clinical and laboratory data. The model is then compared to traditional risk screening and eligibility criteria screening [257]. Li et al. also illustrated various ML applications for lung cancer, specifically in (a) early detection as well as diagnosis, (b) immunotherapy, and (c) treatment and survival prediction [258]. Currently, a Support Vector Machine (SVM) is working on a trending classifier. For one study, lung cancer patients were identified utilizing an SVM classifier according to their symptoms, and the Python programming language was also used to advance the model’s execution. The performance of the SVM model was assessed utilizing a number of different metrics. The suggested model was contrasted with the SVM and SMOTE techniques currently in use. Compared to the new approach, the current ones yield a 98.8% accuracy rate [259]. Some ML algorithms were employed for lung cancer via feature selection. Whitney et al. and Liang et al. chose the best indicators for model training using the least absolute shrinkage and selection operator (LASSO) technique [260,261]. To obtain better findings, a few investigations [262,263] integrated the -omics signatures with clinical markers. Many algorithms, including K-nearest neighbors (KNN), Naïve Bayes, SVMs, decision trees (DT), logistic regression, random forest, linear discriminant analysis, gradient boosting, and neural networks, have shown their capacity to successfully identify and classify various lung cancer patterns utilizing these specific types of tumor -omics signals. Li et al. even tabulated the studies performed by different researchers using different ML models and classifiers for the early detection and diagnosis of cancer [258]. Figure 9 summarizes all the applications of ML algorithms to date in lung cancer management.

### 4.2. Colon Cancer

**Figure 10:**
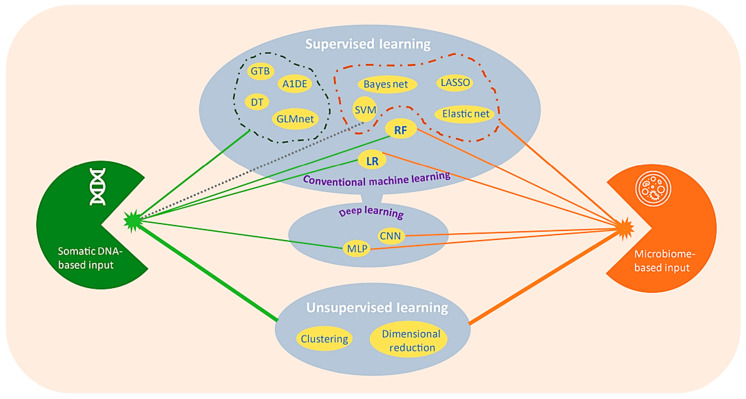
Commonly used ML classifiers in colon cancer [266].

The primary method for diagnosing colon cancer is colonoscopy, which is a surgical procedure, and diagnostic resources are typically limited. Additionally, making a diagnosis is a difficult procedure that involves a chain of interactions between the patient, the original consulting doctor, and the available healthcare technologies [264]. There are a number of datasets that are currently used in colon cancer diagnosis. To prevent overfitting, a technique for minimizing the number of features (genes) unrelated to the target illness is required. The likelihood of recovery from cancer is increased with detection at an early stage. In a study, Loey et al. chose the vital features from the provided data patterns using information gain (IG) and incorporated them into an ML system. The grey wolf optimization technique is then used to minimize the number of chosen features. A vital performance metric in illness diagnosis, classification accuracy, was used to assess it. In order to classify cancer types, an SVM classifier was used [265]. Figure 10 mentions the commonly used ML classifiers in colon cancer.

### 4.3. Pancreatic Cancer

There are certain instances in which it is challenging to separate the diagnostic results of pancreatic cancer from other benign pancreatic disorders. Given the varied treatment consequences, accurate diagnosis in these situations is essential [267]. The capability of ML models like K-nearest neighbor (k-NN), artificial neural networks (ANNs), and SVMs to extract distinctive signatures from medical scans that could potentially be utilized in the diagnosis of pancreatic cancer has been studied [268,269]. Some widely used classifiers for the diagnosis of pancreatic cancer include LR, RF, and SVM. The radiomics and RF classifier in research using CT scans from 190 patients with pancreatic ductal adenocarcinoma and 190 healthy subjects obtained 99.2% correctness in binary categorization [270]. Schultz and co-workers used microRNA panel recognition as a classifier for pancreatic cancer, utilizing two brand-new microRNA panels that combine 4 or 10 microRNAs from whole blood to detect pancreatic cancer [271].

### 4.4. Glioma

Glioma research using ML techniques has grown significantly in recent years. In one study, three datasets with three MRI sequences that are T1-weighted (T1W), T2-weighted (T2W), and fluid-attenuated inversion recovery (FLAIR) were created to improve the distinguishability between low-grade and high-grade gliomas. The same assessment values were obtained with six identical chosen features using the RF and LASSO methods [272]. In another study, a novel deep feature fusion-based multiclass brain tumor classification system was proposed by Kibriya and the team. As a preprocessing phase, a min–max normalization algorithm with data augmentation was applied. From transfer learning architectures like AlexNet, GoogleNet, and ResNet18, deep CNN features were taken out and combined to form one feature vector. On this feature vector, a classifier using SVM and KNN models was employed. The proposed framework was trained and assessed using a dataset of 15,320 MR images. The study’s findings show that the fused feature vector performed better than the component vectors. The new method also performed better than the existing methods, obtaining 99.7% accuracy [273]. Takahashi et al. attempted to gain preoperative (before surgical resection or biopsy) MR images from 951 adult diffuse glioma patients from 10 facilities in Japan. Of the 951 patients, 673 cases had at least one series of preoperative digital MR pictures, and 544 instances matched their criterion. The tumor tissues taken from these individuals were subjected to sequencing analysis after thorough clinical data collection. These pictures are referred to as the Japanese Cohort (JC) dataset. This is the largest glioma imaging collection with access to clinical data as well as genetic/epigenetic profiles after the further division of the 544 subjects, which were classified into three groups. Compared to the Multimodal Brain Tumor Image Segmentation Benchmark (BraTS) dataset, the number of patients included in the JC dataset was 1.6 times higher [274].

### 4.5. Skin Cancer

The difficulty of making an early diagnosis of melanoma, even by professionals, is a serious issue. As a result, doctors may find it useful to use a procedure that simplifies its diagnosis [275]. The use of processing images and machine vision technologies for various medical imaging applications has been growing rapidly in the last ten years. Currently, non-melanoma and melanoma skin cancer diagnosis and surgical planning employ supplementary imaging technologies most commonly [276].

A group of researchers created the PH^2^ dataset to aid in the study of classification as well as segmentation techniques [277]. PH^2^ is commonly employed as a dataset for evaluating skin disease detection algorithms. To dynamically diagnose and segment the dermoscopic pictures in PH^2^, for instance, the SegNet framework was employed, and the classification accuracy was ultimately 94% [278]. It includes 200 color dermoscopy pictures (768 × 560 pixels in size) of three different skin conditions, namely melanomas, atypical nevi, and common nevi. Additionally, it has comprehensive medical annotations, including pathological diagnosis and the findings of lesion segmentation. BCN20000 is used to examine skin cancer lesions in challenging-to-diagnose areas, such as the mucous membranes and nails. In total, 5583 skin lesions and 19,424 dermoscopic pictures captured using high-resolution dermoscopy make up the BCN200005 dataset. From 2010 until 2016, they were all united together. The collector also used a number of computer vision algorithms to clean up the photographs of background noise along with other distractions [279]. Algorithms based on ML for skin cancer diagnosis are rapidly being developed using publicly available datasets of skin imaging data, such as those maintained by the International Skin Imaging Collaboration (ISIC) archive [280,281]. ML algorithms, nevertheless, are prone to overfitting with training data from restricted populations that are frequently curated retroactively, and their generalizability is significantly impacted by the participants and training pictures that are subject to selection bias [282]. Neural networks are one of the most widely utilized methods for image analysis. Deep neural networks, such as convolutional neural networks (CNNs), are often utilized in ML for healthcare purposes [283]. It is recommended to use a variety of ML approaches in a computerized decision framework to recognize melanoma skin lesions accurately and automatically. In the current study, the Clinical Proteomic Tumour Analysis Consortium Cutaneous Melanoma (CPTAC-CM) dataset and the International Skin Imaging Collaboration (ISIC 2019) dataset are both used [284,285].

### 4.6. Oral Cancer

There are several behavioral characteristics that oral cancer might exhibit. Early detection and precise prognostic prediction are crucial for correct and efficient oral cancer care. To achieve this, ML, a branch of AI, has been hailed for its capacity to change cancer management through enhanced diagnostic accuracy and the forecasting of outcomes [286]. The three factors crucial for early diagnosis and prognosis were discovered to be advantageous in the ML technique. These are more accurate forecasts of cancer susceptibility, recurrence, and survival [287], which increase survival rates by effectively managing patients [288,289,290]. Due to its viability and numerous benefits, the ML technique will continue to be used to identify oral cancer [291], forecast oral cancer recurrence, identify occult node metastases [292,293], and estimate oral cancer survival rates [294]. 

A study developed a 3DCNNs-based image processing system and compared it to a 2DCNNs-based algorithm for the early detection of oral malignancies. The same hierarchical structure was used to build the 3D and 2D CNNs to categorize oral cancers as benign or malignant. The results indicated that, for the discriminating of oral cancer lesions, 3DCNNs with dynamic features of the enhancement rate picture outperformed 2DCNNS with a single enhancing sequence. These findings suggest that the spatial dynamics and characteristics collected from 3DCNNs may guide the design of a future CT-assisted diagnostic system [295]. A research team created an ANN model that uses information on risk factors, overall health, and clinic-pathological characteristics to forecast a person’s likelihood of acquiring oral cancer. The ML model for forecasting was created using the well-known data mining technique, ANN. The model was constructed utilizing a total of 29 variables related to the patients. The training dataset had 54 (75%) instances, whereas the testing dataset contained 19 (25%) cases, and the prediction accuracy for oral cancer was 78.95%. Based on the datasets, ML techniques may aid in the detection and diagnosis of oral cancer [296]. 

According to López-Cortés et al., SVMs, ANNs, and LR were the primary algorithms used in the context of medical applications for oral cancer, making up 87.71% of all studies published on this subject. SVMs are one of the ML algorithms with the broadest range of usage. A total of 45.45% of all studies for diagnosis and prevention and 63.63% of all studies for malignant oral lesions (precancer) are focused on SVMs. Additionally, with 43.75% of the prognostic workload, LR was the method most commonly utilized. With algorithms like SVMs, ANNs, LR, and CNNs, ML is a potent technique that can accurately forecast outcomes to assist in diagnosis and prevention, prognosis, possibly malignant oral lesions (pre-cancer), treatment, and quality of life. It is essential to remember that not all existing algorithms are instinctual. The outputs produced by ANNs and SVMs are nonlinear and perplexing. In this way, unlike DT, which reveals the set of rules behind the classification, medical professionals frequently lack confidence in the outputs of clinical decision support systems because it is unclear how the algorithm generates the classification result [297].

## 5. MM Techniques in Specific Types of Cancer

### 5.1. Tumor Growth

The early spatio-temporal modeling of avascular tumor maturation discusses the changes in the structure and size of 3D multicellular spheroids with regard to manipulation in the ambience of culture [298,299]. As we have mentioned before, MMs in the present day are more complex, and most of them are a continuum of the early established MMs [104,105]. Let us assume that spheroids are radially symmetric and their development is modulated by only a diffusible growth factor, which is either externally supplied, e.g., oxygen, or internally produced, e.g., tumor necrosis factor (TNF). The dispersal of growth factor in the spheroid modulates its functional activity if the cell does not reach the point of regression, death, or else. With the integration of these contributory activities on the tumor volume, the following equation can be written. The equation connects the evolution time of the tumor radius, denoted as *R*(*t*), to the growth factor distribution in the spheroid, denoted as *c*(*r*,*t*), as follows:

(15) $$\frac{dR}{dt}=\frac{1}{R^{2}\;}\int_{r=0}^{R}F\left(c\right)r^{2}dr$$

Here, *c* = growth factor

F(c) = The influence of c on the net cell growth rate at each point in the spheroid.

Suppose c is oxygen or glucose. So, it can be assumed that F increases along with c, and it approaches a max value for greater values of *c*. Hence, the equation for the spatial distribution of *c* is as follows:

(16) $$\frac{\partial c}{\partial t}=\frac{D}{r^{2}}\;\frac{\partial }{\partial r}\;\left(r^{2}\frac{\partial c}{\partial r}\right)-g\left(c,R\right)$$

Here, *D* = Diffusion coefficient of *c*

*g*(*c*,*R*) = Local consumption rate of *c*

These equations can also be used to predict the spheroid structure. The function of *F*(*c*) is basically cell-growth-specific. It might be dependent upon cell proliferation, quiescence, or death [299,300].

In 1987, after identifying the retinoblastoma 1 (Rb1; a tumor suppressor gene), the two-hit hypothesis by Knudson was confirmed. This theorem has helped researchers to characterize the inactivation of other tumor suppressor genes, for instance, the adenomatous polyposis coli (APC) gene in colon cancer and tumor protein 53 (TP53), which has been mutated in more than 50% of human tumors [301]. Currently, the ‘hallmarks of cancer’ model [29] is being used to sequence the mutation and observe its evolution timing, along with the influence of environmental factors on the progression of tumors [302,303,304]. 

There comes another MM model that regards tumor angiogenesis, which was proposed by Balding and McElwain [305,306,307]. This model focuses on the tumor angiogenesis factor along with blood capillary end and vessels that are denoted as *a*, *n*, and *b*, respectively. To represent a one-dimensional model, where *x* represents the distance between the focal point of a tumor and the vasculature, the equation for *a*(*x*,*t*) is as follows:

(17) $$\frac{\partial a}{\partial t}=D_{a}\;\frac{\partial^{2}a}{\partial x}-{µ}_{a}a$$

Here, $D_{a}$ = Presumed constant tumor angiogenesis factor diffusion coefficient

${µ}_{a}$ = Natural decomposition rate of tumor cell

Since it has already been presumed that a tumor will produce angiogenesis factor at a constant rate, the concentration of angiogenesis factor at the outer edge of the tumor is also maintained at a constant value. The end of blood capillary is supposed to emerge from existing blood vessels and tips at a rate that increases proportionately with the level of tumor angiogenesis factor to start chemotaxis using the upper spatial gradients of angiogenesis factor, and, thus, create end-to-end anastomosis. By combining these interactions, the equations for *n(x,t)* is as follows:

(18) $$\frac{\partial n}{\partial t}=-\chi \;\frac{\partial }{\partial x}\;\left(n\frac{\partial a}{\partial x}\right)+\left(\lambda_{nb}b+\lambda_{nn}n\right)\left(\frac{a}{k_{\partial }+a}\right)-\mu_{n}n-\vartheta_{n}n^{2}$$

Here, χ = Chemotaxis coefficient

$\lambda_{nb}$ = The rate at which blood capillary emerges from existing blood vessels

$\lambda_{nn}$ = The rate at which an end of blood capillary emerges from existing blood vessels

$\mu_{n}$ = Net rate at which blood capillary tips expire

$\vartheta_{n}$ = The rate at which capillaries form end-to-end anastomoses [307,308].

After the migration of capillary vessels near the tumor, the edge of the capillary extends towards it to connect to new vessels and leave the residue behind. Now, as per this speculation, the equation for the blood vessels *b*(*x*,*t*) is as follows:

(19) $$\frac{\partial b}{\partial t}=\chi^{n}\frac{\partial a}{\partial x}-\mu_{b}b$$

Here, $\mu_{b}$ = the rate at which blood vessels revert

Mathematical simulations of Balding and McElwain’s model and its ensuing additions reveal varied architectural attributes of tumor angiogenesis that include the acceleration of vasculature development in the direction of the tumor and peak density of the capillary tips, foregoing the peak density of blood vessels. By using this model, antiangiogenic treatment for counterpoising tumor angiogenesis factor was compared to other treatments that inhibit endothelial cell proliferation or the chemotaxis of cells [305,309,310]. Furthermore, intricate MMs of the genetic regulatory network (GRN) and principal signaling pathways are widely being applied in the study of angiogenesis [311,312]. In 2009, Wu and team applied a compartment model with the purpose of mathematically proving that an innately derived, soluble vascular endothelial growth factor receptor 1 (VEGFR1) fails to notably block the VEGF signaling pathway, and came up with a conclusion that if any signal limiting effect is observed, then the heterogeneous blood flow might be the reason [313]. 

Swanson’s reaction–diffusion model gives us insight into the growth and invasion of glioma [314,315,316]. Tracqui and co-workers proposed a spatio-temporal model which tells us about the uncontrolled proliferation of cancer cells, along with their capability for metastasis [317]. Harpold et al. simplified this spatio-temporal model based on the fact that glioma cells never take part in metastasis outside the brain. Suppose, as per mm^3^, that the concentration of tumor cells is *c* = *c*(*x*,*t*) at time t and location *x*, where brain domain B is enclosed. So now, as per the model, the tumor cell proliferation and net diffusion can be described as follows [318]:

Rate of change in concentration of glioma cells = Net glioma cell dispersion + Net glioma cell proliferation (20)

Hence, Equation (20) can be mathematically written as follows:

(21) $$\frac{\partial c}{\partial t}=\nabla \;\cdot \;\left(D\left(x\right)\nabla c\right)+\rho c\;$$

(22) $$⇨x\in B,\;t\geq 0,c\left(x,0\right)=c_{0}\;⇨n\cdot \nabla c=0\;on\;\partial B$$

Here, *D* = Spatially resolved diffusion coefficient at mm^2^/year

$c_{0}$ = Initial distribution of tumor cells

ρ = Net rate of proliferation per year

n·∇c = Zero flux boundary condition

∂B = Boundary of brain domain

Equation (22) describes that, at the boundary of the brain domain, a zero-flux boundary condition (*n* bullet dell *c* = 0) inhibits tumor cells from leaving the brain domain [318,319,320,321].

### 5.2. Treatment

MM is also being utilized in optimizing therapeutic protocols that involve combining chemotherapy, radiotherapy, and surgery. Moreover, it is used in advanced cancer treatment development [322]. In 1982, a model was proposed by Barendsen based on a linear-quadratic model for the total dose calculation of a treatment regimen, which is presently known as the biologically effective dose (BED) [323]. Previously, this formula used to be known as the extrapolated response dose, which was changed by Fowler later on in 1989 [324]. It is widely applied to calculate fractions (doses) of external radiation therapy in the treatment of cancer [325,326,327]. The equation for radiational dose calculation is as follows:

(23) $$BED=-\frac{\ln\left(\sigma \right)}{\alpha }=D\left(1+\frac{d}{\alpha /\beta }\right)\;\;=nd_{1},d_{2},d_{3}\ldots \ldots \ldots d_{n}\left(1+\frac{d}{\alpha /\beta }\right)$$

Here, *n* = Number of fractions

*d* = Radiational dose per fraction

*D* = Total dose delivered

*α*/*β* = Fractionation sensitivity

Hence, we can say that BED = total physical dose × relative effectiveness [325,328]. Generally, the value of *α*/*β* is tumor-site-dependent [329,330,331], because it is a vital factor in determining the radiobiological properties of cells [332,333]. 

In 1988, Jain and his colleague proposed a model to find out the reason behind the lower delivery of anti-cancer drugs in the case of vascular tumors. Their model concluded that the uneven regional perfusion and blood flow and the higher pressure of interstitial fluid prevent the delivery of drugs to vascular tumors, which was later confirmed in 2004 by Owen et al. [334,335]. Rockne et al. combined the aforementioned linear-quadratic model with Swanson’s reaction–diffusion model to compare the effectiveness of radiational dose distribution and different dose timings [321]. Another mathematical approach was conducted by Siegmund and co-workers, who analyzed DNA methylation patterns so that genetic evolution could be blocked at several sites in the tumor cells of colorectal cancer [336]. 

In another work, Swanson and team implemented the reaction–diffusion model on the magnetic resonance imaging (MRI) data of glioma patients to predict the possibility of glioma regeneration after surgery [337].

MMs are also being used in cell signaling pathways, drug designing to find new targets, and determining the pharmacokinetic–pharmacodynamic effects of a new anti-cancer molecules [311,313,338]. Talking about targeted drug delivery in cancer, another MM was developed by Panetta that demonstrates the impact of “cell-cycle-specific” anti-cancer drugs (paclitaxel was used here) on both cancerous cells and normal tissues. To obtain this, this model considered two types of cells: (a) proliferating cells (these are sensitive to the paclitaxel) and (b) quiescent cells (these are resistant to paclitaxel) [339].

In 2005, Basse et al. developed an MM for a human tumor cell line that has not been perturbed by anti-cancer treatment [340]. The modeling was performed on the basis of the different phases of the cell cycle, that is, the gap 1/growth phase 1 (G_1_ phase), gap 2/growth phase 2 (G_2_ phase), synthesis (S), and mitosis (M). In this model, Basse and team assumed that the cell’s dynamics are regulated by the system of partial differential equations (with *t*, *τS* > 0 and 0 < *x* < *X*) as follows:

(24) $$\frac{\partial G_{1}}{\partial t}\left(x,t\right)=4bM\left(2x,t\right)-k_{1}G_{1}\left(x,t\right)$$

(25) $$\frac{\partial \bar{S}}{\partial \tau_{s}}\left(x,t;\tau_{s}\right)+\frac{\partial \bar{S}}{\partial t}\left(x,t;\tau_{s}\right)=D\frac{\partial^{2}\bar{S}}{\partial x^{2}}\left(x,t;\tau_{s}\right)-g_{S}\frac{\partial \bar{S}}{\partial x}\left(x,t;\tau_{s}\right)$$

Here, 0 < *x* < *X* = Relative DNA content

*t* = Time

$\tau_{s}$ = Amount of time cells spend in the synthesis phase

$\bar{S}\left(x,t;\tau_{s}\right)$ = Number density of cells that have been in the synthesis phase for τ_S_ hours at time t

*G*_1_(*x*, *t*) = Number density of cells in gap 1 phase

*D* = Dispersion coefficient

$g_{S}$ = Average growth rate of DNA in the synthesis phase

There are two transitions in the cell cycle. One is the transition between gap 1 and the synthesis phase, and the hourly rate of this transition between these two phases can be denoted as k_1_. Another one is the transition between gap 2 and the mitosis phase, and the hourly rate of this transition between these two phases can be denoted as k_2_. If from the mitosis phase to the gap 1 phase, cells get divided with a rate b per hour, then, as per the prediction that cells spend a fixed time (can be denoted as *T_S_ h*) in the synthesis phase, the equation will be as follows,

(26) $$\frac{\partial G_{2}}{\partial t}\left(x,t\right)=\bar{S}\left(x,t;T_{S}\right)-k_{2}G_{2}\left(x,t\right)$$

(27) $$\frac{\partial M}{\partial t}\left(x,t\right)=k_{2}G_{2}\left(x,t\right)-bM\left(x,t\right)$$

Here, *b* = Division rate

*G*_2_(*x*, *t*) = Number density of cells in gap 2 phase

*M* (*x*, *t*) = Number density of cells in the mitosis phase

After staying in the synthesis phase for $\tau_{s}$ = *T_S_* hours, cells move to the gap 2 phase. Primary dispersals at time *t* = 0 in the gap 1, synthesis, gap 2, and mitosis phases are the capricious positive functions *G*_1_(*x*, 0) = *G*_10_(*x*), $\bar{S}$(*x*, 0; *τ_S_*) = $\bar{S}$_0_(*x*, $\tau_{s}$), *G*_2_(*x*, 0) = *G*_20_(*x*), and *M*(*x*, 0) = *M*_0_(*x*), respectively (where, 0 < *x* < *X*). Now, at the zero-flux boundary conditions in the synthesis phase, Equation (24) will be as follows:

(28) $$D\frac{\partial \bar{S}}{\partial x}\left(0,\;t;\;\tau_{s}\right)-g_{S}\bar{S}\left(0,\;t;\;\tau_{s}\right)=0\;\;t,\;\tau_{S}>0$$

(29) $$D\frac{\partial \bar{S}}{\partial x}\left(X,\;t;\;\tau_{S}\right)-g_{S}\bar{S}\left(X,\;t;\;\tau_{S}\right)=0\;\;t,\;\tau_{S}>0$$

The primary stage (*τ_S_* = 0) for Equation (25), which represents those cells coming from the gap 1 phase (where, *t* > 0, 0 < *x* < *X*), will be as follows:

(30) $$\bar{S}\left(x,t;\;\tau_{s}=0\right)=k_{1}G_{1}\left(x,\;t\right)$$

Hence, for any given time t_0_, the distribution of the DNA of the cells can be attained by adding the profiles in each phase = $G_{1}\left(x,t_{0}\right)+\int_{0}^{T_{S}}\bar{S}\left(x,t_{0};\;\tau_{S}\right)d\tau_{S}+G_{2}\left(x,t_{0}\right)+M\left(x,t_{0}\right)$ [340]. DNA profiles acquired by using these MMs on a human tumor cell line, which have not been perturbed by anti-cancer therapy, show a phenomenon called steady DNA distributions (SDD), regardless of the primary dispersal, which is the feature of another MM mentioned by Arino [341]. 

### 5.3. Interconnection between ML and MM

**Figure 11:**
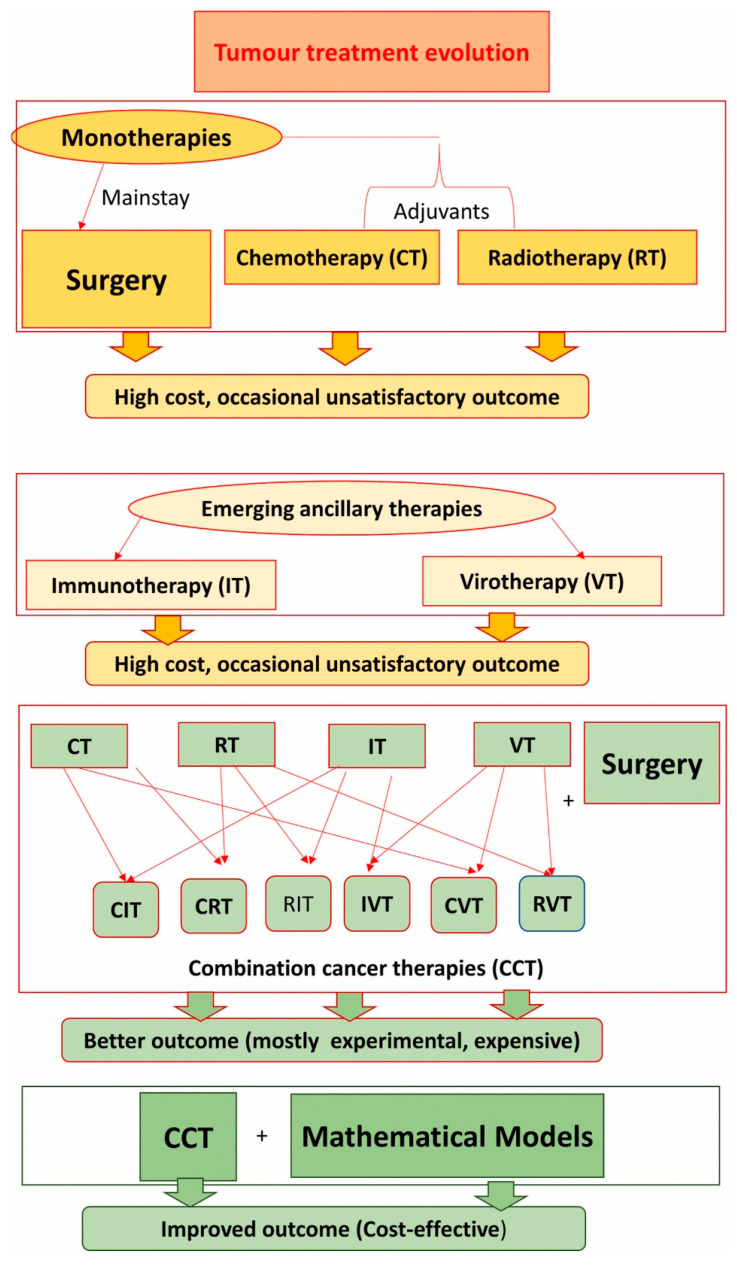
Schematic representation of achieving worthwhile therapeutic outcomes using a combination of ML and MM techniques in cancer treatment [351].

When it comes to any advanced technology, ML and MM are intertwined [342,343]. Machines learn from trained datasets with the help of ML. This learning process is attained through varied mathematical symbols, expressions, and logical equations. Hence, this fact self-describes the relationship between ML and MM. MM supplements an established ML model to provide better outcomes, however, sometimes, the opposite also happens [344,345,346]. For example, during the recent COVID-19 outbreak, when researchers were busy producing different strategies in every possible area of science to assist the worldwide situation, Liu and colleagues used a neural network (ML) to solve the limitation of the SEIR model (MM). Based on the assumption of the SEIR’s inflexion point, they used a neural network to acquire an accurate prediction and further refined the fitting at certain time points [347]. In 2014, Bezak et al. published an article where they used MM to map a robotic hand to overcome the shortcomings of their applied ML model [348]. Additive manufacturing (AM) is the process of adding layers upon layers to form a 3D structure [349]. Powder bed fusion (PBF) is one type of additive manufacturing process. Baturynska et al. used a combination of MM and ML optimization methods for the purpose of the optimization and evaluation of the parameters of this PBFAM process [350]. Similarly, numerous approaches have combined these techniques to achieve a more accurate result in the field of cancer research. Figure 11 shows that researchers achieved better therapeutic results when they used a combination of ML and MM, which is, most importantly, cost effective.

In terms of the benefits of combining ML and MM, telemedicine is a good case in point. Telemedicine can make the healthcare system more available and safe in case of infectious diseases, for example, COVID-19 [352,353]. Sirintrapun and Lopez stated teleoncology as a strategy for improved accessible care with lower treatment and maintenance costs [354]. Moreover, in the case of rural populations, oncologists are not required to travel to the location to provide care [355]. Doolittle et al. [356,357] and, recently, other researchers [358,359,360], also demonstrated the patient satisfaction, improved clinical efficacy, and cost-effectiveness of this telemedicinal approach from their experience in cancer patient care [354]. Shalowitz and Moore mentioned in their recently published article that telemedicines have the potential to reduce or eliminate geographical barriers in providing improved-quality care to cancer patients. They also mentioned their existing applications in cancer care, which are, (a) pre-diagnosis, (b) pre-treatment, (c) treatment, and (d) post-treatment monitoring, at different phases [361]. A study was conducted by Yunus et al. on 217 patients to evaluate the safety, quality, and cost for cancer patients receiving rural telehealthcare (134 patients) in comparison to “routine” urban healthcare (83 patients). The resulting data showed that the telemedicine visits were as good as or better compared to in-person visits, with a 95% patient satisfaction rate [358]. 

Wearable technology and at-home smart electronics offer a new way to control health outside hospital settings [362,363,364]. These gadgets have the capacity to gather substantial volumes of meticulous data on patient health conditions, which are utilized by ML algorithms to recommend one-time actions, adjustments to daily routines, or referrals to a doctor for evaluation and testing. Sensors for mobility, pulse, respiration rate, body temperature, blood pressure, oxygen levels, and other biometrics are becoming a common feature of wearable technology [365,366,367,368,369]. In the future, wearable and sound sensor data will probably be utilized to find novel biomarkers, possibly by merging data from various types of devices [130,370,371]. The continuous monitoring of a person’s behavior and bodily functions via wearable and home gadgets, along with readouts from routine blood tests, are features of health management. By modifying individual-level models with information gathered for each individual, a customized model of essential functions and activities will be created. The ability to construct individualized outlines and detect their shifts, which may signify a change in health condition, is a major benefit of this technique. ML-based applications will monitor people using customized models for any deviations from the normal and alert them when a change requires consulting a medical specialist. In order to create models using wearable data, both conventional supervised learning and deep learning are likely to be included [372,373,374,375,376].

## 6. Challenges of ML and MM Approaches in Cancer Prognosis and Therapy

Over the decades, a wide range of FS algorithms have been extensively used in the prognosis and prediction of ailments. The majority of published work discusses the use of ML techniques to simulate the development of cancer and find relevant aspects that are then used in a categorization system [15]. The major challenge in developing an ML tool starts from the very beginning during the training dataset preparation. Missing data are a common phenomenon in this case. The factor that usually leads to missing data is a participant not answering all sections in a questionnaire, maybe due to, (a) time shortage, (b) insufficient knowledge to respond to a particular question, (c) failure to understand the questions, (d) not feeling the desire to respond, and (e) finding certain questions embarrassing to answer. In addition, the quantity of missing data increases with the inclusion of multivariate data in a dataset. The productivity of ML tools is severely influenced by such missingness in datasets. Researchers always try to reduce missing values because of their unavoidable existence, which is a concern for empirical scientists in varied disciplines [377,378,379,380,381]. 

**Figure 12:**
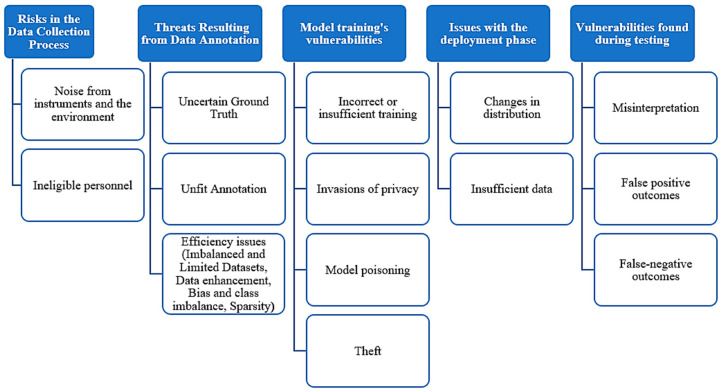
Challenges of ML application and cyber threats in healthcare.

Another issue is that patient selection bias in ML models might result in subpar performance and inaccurate predictions in future unanticipated circumstances, because a large proportion of AI models are trained using retrospective, observational data [382]. A patient’s desire to maintain the privacy of their health and social details should be respected, so patients’ information must thus be meticulously gathered to obtain the data required to create models [383]. ML-based diagnostic systems could also be biased and prone to mistakes. Due to this, the outcomes from these models cannot be relied upon blindly, since they could potentially harm patients if there is any incorrect information. Health practitioners need to have a thorough grasp of the process of reviewing data sources, model construction, and algorithm formation in order to build intended ML models to predict outcomes. To successfully implement and utilize ML-based prediction models, cooperation between ML specialists and health professionals is required [384]. Figure 12 illustrates an in-depth understanding of ML challenges and categorizes them into different sectors. As mentioned before, these challenges could be sidestepped with the consultation of ML specialists to improve ML algorithms by frequent modification and upgradation. Ethical considerations while developing ML algorithms should be taken into account as well to overcome the issue of patient privacy [385]. 

MM coupled with well-designed in vitro experiments provides a way of cancer treatment investigation that may cause a reduction in the number of animal studies. We can now understand cancer from several angles with the assistance of MM. The processes involved in creating an MM are: (a) selecting a real-world issue; (b) simplifying biological phenomena; (c) creating a mathematical quantification; and (d) running numerical simulations [386]. The accuracy of these models is discerned only when the results are within the parameters. A significant difficulty for computational oncology is obtaining physiological-based findings for combination chemotherapy and antiangiogenic treatment [387].

### 6.1. Data Quantity

Any data-driven project must consider the quality and quantity of the data required for ML models. Data scientists may accurately assess the project’s scope, schedule, and viability by knowing the minimum dataset size needed. The kind of issue being handled, the model complexity, the quality and correctness of the data, and the accessibility of labeled data are all considered when estimating the volume of data required for an ML model [388,389,390,391]. Data amount estimation can be handled via statistical methods for large datasets and the rule of thumb approach for smaller datasets [392,393]. Applying the 10 times rule is the most typical technique to determine whether a data collection is enough. According to this principle, a model should include 10 times more input data (i.e., instances) than degrees of freedom. Degrees of freedom often refer to variables in the specified data collection. This could also mean that input data need at least 10 times as many data points (rows) as there are features (columns) in the dataset when using ML [394].

For an ML model to be adequately trained for a medical technology solution, data accessibility by itself is frequently insufficient. In healthcare initiatives, the accuracy of the data is crucial. Research in this area is challenging because of heterogeneous data formats. The varied forms of data from laboratory tests, medical pictures, vital signs, and genomes make it difficult to apply ML algorithms to all data concurrently. The widespread availability of medical datasets is another problem in making a dataset with good-quality data [395,396,397,398].

Applications for ML in healthcare span from completely autonomous AI for cancer detection to non-autonomous mortality estimates to help allocate resources [399]. It may be quite challenging to strike a balance between information quantity and contemporaneity. Modifications are frequently made to the information gathered and the testing procedures. This implies that the models that use data gathered over a lengthy period of time need a method to handle these modifications and to compare normal values and ranges. A model should be receptive rather than generalizable, recognizing incoming data that differ from the training or test set. A model with suitable governance of versions and data needs that can be modified and revalidated might prove more beneficial [400]. The fact that even DL, which is a subset of ML, is data greedy constitutes one of the biggest obstacles to its implementation. DL uses a lot of data to forecast a group of unobserved data and understand how features behave during training [401]. In order to avoid overfitting, modern deep neural networks generally feature numerous parameters that can be trained and need a corresponding amount of labeled pictures during training [402]. 

### 6.2. Ethical Consideration

Even though the methodology of how ML and MM work is known, ethical considerations need to be put into play when working with them. AI algorithms have special ethical and legal issues that restrict their widespread use and reproducibility, particularly their natural bias when developed on datasets that preferentially omit underrepresented people. Furthermore, algorithms must show dependability, validity, and transparency [403]. The security and confidentiality of medical information, biases in the data employed when constructing the model, trustee connection, dispute, a lack of duty or liability, relationships between doctors and patients that might alter the autonomy of patients, and a violation of the independence of patients, along with criticism by peers were among the ethical issues covered in research that attempts to offer a systematic evaluation of the moral and societal effects of ML models on the therapy of oral cancer [404]. Despite its promise, utilizing ML for forecasting mortality in the pediatric ICU raises ethical questions about bias, trust, and the effect on treatment. Forecasts are affected by a dataset’s lack of variety or correctness. ML is dependent on “learning” from large datasets. Simulations that depend on data regarding ICU fatalities, nonetheless, do not take into account the possibility of death following hospitalization, leading to a sort of reverse survivorship bias. A prejudiced algorithm may exist itself. In the event that providers grow reliant on AI, backup procedures must be in place in case of system failure or during routine upgrades. The black box phenomenon refers to some algorithms for forecasting that are so complicated that it is impossible to understand how judgments are made. If the models are not carefully examined for their safety and efficacy, this paucity of transparency can result in skepticism and may impair physician and patient acceptability, as well as the usage of such technologies [405]. How doctors and healthcare institutions use the data produced by risk estimation algorithms raises a separate ethical issue. A risk calculator would produce patient-specific risk data in a perfect world, enabling informed permission and collaborative decision making. In a reverse scenario, the dependency on risk calculators may reduce the practice of asking patients about their values, past experiences, and motives when deciding whether or not to implement a future medical intervention. Additionally, doctors may restrict the options offered to patients and their families when the dangers are significant, prioritizing paternalism above regard for autonomy. Additionally, there is a risk that healthcare programs or insurance providers will use risk calculators to select which patients are suitable candidates for particular initiatives, completely avoiding patient–physician decision making or penalizing doctors who function above the limit of an “acceptable” risk spectrum [406].

Ongoing discussion evokes the question of whether ML fits within existing legal categories or whether a new category with its special features and implications should be developed. Although the use of ML in clinical settings holds great promise for enhancing healthcare, it also raises ethical concerns that we need to consider. Four significant ethical concerns need to be resolved for ML in healthcare to realize its potential completely. Important things to take into account include: (a) informed permission to utilize data, (b) safety and transparency, (c) fairness through algorithms and biases, and (d) data privacy. The question of whether ML systems are lawful is controversial politically as well as legally [407,408]. The goal is to support policymakers so they may take prompt action to address the morally challenging circumstances in which mandating ML in medical facilities improves [408,409]. Many legal conversations on ML have been influenced by the limits of algorithmic openness. ML design and governance must now be more responsible, egalitarian, and transparent as ML is used more frequently in high-risk circumstances. The two most crucial components of transparency are information accessibility and understandability. Learning about an algorithm’s operation is usually made difficult on purpose. With machines that function by ML, in case of any issues, the manufacturer or operator will be held responsible for any harm caused [399,408,410,411].

Even though wearable technologies have been used in the medical industry to improve people’s health, they might also pose ethical issues [412,413,414]. While the algorithm and the guidelines are given by humans, the patient information is gathered in accordance with the requirements of the algorithm design. ML imitates how the human brain makes decisions; it is not as intelligent as a real person, which raises moral limitations as well. A significant issue in the medical internet of things (IoT) is the anti-interference capability and security hazards of transmission technologies [415,416,417]. Our understanding of privacy varies depending on the context, and the ethical analysis will differ depending on the person accessing a particular type of data for a certain purpose [418,419].

Regulations play a crucial role in the steady advancement of these technologies. It is necessary to address the problem of relevant regulatory policies by the algorithm developers from all research fields [420,421].

### 6.3. Data Privacy

Data privacy is a concern during ML algorithm data storage and use. Data might be stored in multiple locations to avoid data loss, and thus, there can be risks of data breaches or hacking [422]. Even though people are made aware that a company is gathering information about them, it is not always feasible to refuse to collect such information. Furthermore, privacy safeguards that apply to identifiable personal data in one area may not always be applicable to other places. Such regulatory differences allow public and private organizations to get around data protection laws that they find overly constricting. Therefore, it is even more critical to reconsider the legal conceptions of privacy and property regarding the data practices that enable algorithmic regulation [423,424,425,426]. The primary moral concern is the confidentiality of medical data. A lot of patient medical information is used to build ML models. As a result, the security and privacy of patients are at risk. To solve this problem, it is necessary to notify patients or participants in other studies about the gathering and utilization of their data in order to obtain their informed approval, avoid unlawful proprietary usage of their information, and safeguard their privacy. Approaches for AI-based illness diagnosis have significant challenges in the areas of data protection and privacy: (a) data leaks: AI-based ailments diagnostic technologies are vulnerable to hackers since they contain a lot of private patient information. Unapproved access to or disclosure of patient records might happen as a consequence of an information breach, which could seriously violate privacy rights; (b) sharing of data: AI-based illness diagnostic systems frequently divulge patient information with third parties, including healthcare professionals and research institutes. This may lead to questions about the safety and confidentiality of data as well as possible data misuse; (c) creating data anonymity: Anonymized data may be used by AI-based illness diagnostic systems to safeguard the confidentiality of patients. However, anonymous data continue to be utilized to re-create patients’ identities, which means there is a chance that the information will be abused; (d) storing of data: Systems for AI-based illness diagnosis keep a lot of patient data. The information in question may be kept in several different places and may be subject to security breaches, computer hacking, and data loss; (e) transparency is lacking: Patients may find it challenging to comprehend how their data are utilized and to govern accessibility to their data, since AI-based disease diagnosis methods may not be transparent in how they gather, store, and utilize information concerning patients; and (f) prejudice and bias: Bias and prejudice can have an impact on AI models, which might provide erroneous or unreliable findings, specifically for a particular demographic group [422]. By combining the health insurance portability and accountability act (HIPAA) with the general data protection regulation (GDPR) of Europe [427,428] and the California consumer privacy act (CCPA) [429,430], Bari and O’Neill, 2019, proposed a framework for reconsidering patient data privacy in the era of digital health [431]. One study suggests a brand-new, non-invasive, secure cancer diagnosis technique employing DL. In order to avoid data theft, the information gathered is encrypted before being transmitted across the channel. Correlation, entropy, contrast, structural content, and energy are some of the security metrics that are used to evaluate the effectiveness of the suggested encryption technology. A picture encryption method that uses chaos, discrete wavelet transforms, and bit-plane extraction to transfer data without being modified by hackers or unauthorized access protects the private medical photographs of patients used for cancer detection. The information is received in an encrypted format and then decrypted before being employed for the diagnosis of cancer [432].

Patients must be informed of their privacy rights, the types of PHI that will be shared with third parties, and the purposes for which such disclosures will be made before they are admitted to a healthcare facility. HIPAA now mandates that all patients, regardless of age or gender, receive notice of privacy practices. The patient must sign this form, and a single copy must be stored in the hospital records, which also acts as evidence that the patient received privacy notice. If the patient is unable to sign for whatever reason, the situation must be noted and witnessed. If someone else signs the paperwork, the justification for the signature must be recorded. The healthcare facility is not obligated to continuously inquire about the patient for disclosure of PHI during routine care once they have signed a notice of privacy practice. The note must be updated if the patient’s medical state changes or if they develop new privacy concerns. The patient may request that no friends or family members be allowed to collect their drugs or that the healthcare workers refrain from discussing the patient’s medical condition with them [430,433,434]. Effective data anonymity and security precautions must be implemented to address concerns with data privacy, protection, and governance [435].

## 7. Further Discussion and Future Directions

ML has made life easier due to its quick learning capabilities with almost zero chance of error so that we can focus on other tasks. The objective of ML and MM-driven strategy in healthcare is not only to save time, money, and resources, but also to provide medications to patients more quickly. The main objective is to deliver the same healthcare facilities to people from all socioeconomic categories regarding any ailments, diseases, and accidental cases.

To find causality, we must accurately forecast system dynamics, which motivates the integration of multiscale modeling with ML and MM for biological, biomedical, and behavioral systems. The fundamental question is whether we will eventually be able to use today’s models to detect appropriate biological traits and investigate their interplay in real time. If the progression of disease biomarkers is uncovered and processes are understood from vast datasets, for example, early biomarkers of cancer, it is a highly applicable example of direct translational usefulness. 

Developing data- and theory-driven ways to formulate a mechanistic understanding of the genesis of biological function to explain occurrences at higher levels as a result of collective action on lower scales is the ultimate challenge, to put it more abstractly. In this review paper, we have seen quite a few successful ML, MM, and sometimes combined strategies in cancer prognosis and treatment. These days, the use of MM-driven ML tools has started to revolutionize the cancer management system. There are now several wearable technologies available for tracking physical health. In recent years, the smartwatch market has experienced rapid expansion. The sharp increase in wearable gadget sales indicates customers’ interest in daily monitoring activities. In both clinical settings and laboratory experiments, wearable technology can track physical activity and notify of unpredictable changes in the human body. It is now feasible to remotely track the physiological characteristics of a large number of cancer patients in real time thanks to cutting-edge communications technologies that enable immediate and enormous multidirectional data transfer. Physicians can then obtain these real-time data, subsequently facilitating prompt action. Electronic biosensors have been gradually reduced in size over the past few decades, enabling wearable devices to continuously monitor physiological parameters like the skin temperature, heart rate, respiration rate, oxygen saturation, perspiration, and activity of ambulatory subjects on a 24/7 basis.

For the successful and uninterrupted implementation of these ML-based tools in cancer management, they should be under the systematic vigilance of ML specialists. Systematic flaws in ML-based systems may be found by developers, who may then make appropriate revisions to the model creation process, such as eliminating the offending predictor or using an alternative model. Therefore, vigilance is a must, because ML interpretability might be incorrect or meaningless, especially when the disease is multidimensional, like cancer. Consequently, it may be preferable that a particular ML model be restricted if it is accountable for high risks in cancer patient management.

We have seen how ML and MM are used in various cancer treatment and management forms. This, however, is far from exhibiting their growing potential in the current world. We believe the opportunities for ML and MM are just hitting the tip of the iceberg and future developments are vast. To manage the distribution of healthcare resources, ML healthcare employs a range of techniques, from completely autonomous AI for cancer detection to non-autonomous death estimations. Treatment options using AI and ML are expanding, and they range from robots that are located in communities to virtual psychotherapists. 

High-performance computing is an aspect of computing that can process massive amounts of data, and it has recently become widely employed in many different industries. Medical investigators can learn more about probable root causes and therapies for illnesses by using large-scale data analysis techniques based on high-performance computing. Researchers in medicine can learn the processes of cancer incidence and progression and, hence, create more efficient cancer therapies by examining large-scale genomic datasets. They are able to analyze and interpret picture data more quickly due to high-performance computing, which also increases the precision and efficiency of medical diagnoses. The functioning of human organs, along with disease processes, may be simulated and examined by medical scientists with the use of high-performance computing. Medical professionals may more correctly investigate human physiology and disease processes and create more efficient therapies by using MM based on high-performance computers.

With the help of virtual reality (VR) innovations, patients may gradually adjust to and recover from their psychiatric illnesses in a secure environment. Although the combination of MM with VR technology is not quite there yet, it has demonstrated an extensive spectrum of possible applications. With the use of MM and VR devices, medical professionals will be able to improve how they diagnose and treat patients in the future. They can be utilized to alleviate cancer-treatment-related side effects such as persistent pain and post-surgical discomforts.

One study stipulated that ML does not anticipate displacing radiologists any time soon. Instead, these methods are anticipated to assist radiologists, streamline radiology operations, and raise the diagnostic reliability of radiologists. The use of ML techniques may make it easier to spot linkages and patterns that would often escape human observation. Many AI programs are currently being built on straightforward tasks that pose no difficulty for humans. If efforts are concentrated on jobs that are difficult for radiologists to perform, these AI tools may be of more benefit. Perhaps the earliest medical specialties to apply ML algorithms may be imaging for diagnostic purposes, but other disciplines, including pathology, cardiology, dermatology, and gastrointestinal, all have potential applications [436]. 

An area of biotechnology known as “gene editing” uses instruments like CRISPR/Cas9 to alter gene sequences accurately. With the use of this technology, people with genetic disorders may be able to regain normal function by targeting and altering certain genes. Additionally, technology for editing genes can be utilized to treat conditions affecting the immune system, cancer, and cardiovascular disease. The majority of present therapies are created based on typical results and cannot be specifically adjusted to each patient’s particular circumstance. It would be a significant advancement if personalized treatment regimens could be developed using ML and MM algorithms to take into account each patient’s genetic makeup, medical background, and other clinical data.

## 8. Conclusions

Applications of ML and MM in the overall management of different cancers are undeniably powerful these days, and trendy as well. First, advancements in the different cancer biology research require initial MMs to investigate the potential of hypotheses. If researchers attain a positive outcome, they proceed to further investigation via ML models, where again, MMs are also sometimes required to make the experimental outcome more accurate. If researchers obtain a positive outcome from this experimental stage, they proceed further to laboratory-based experiments. By using MM and ML techniques, researchers can rapidly predict cancer susceptibility, recurrence, and survival rate along with the best possible treatment combination as well. Before scientists used to directly execute laboratory experiments, however, this was a time-consuming, costly, and tiring. This is why, before heading to the lab, multiple computational steps are now performed first. This combinatorial approach of ML and MM makes cancer management feasible, cost-effective, and more accurate than before.

## Figures and Tables

**Table 1:** Features of ML algorithms used in drug discovery and design.

SN	ML Tool	Description of Algorithm	Features of Algorithm	Reference
1.	Drug finder	In silico virtual screening (VS)	Used to validate the screening platform along with its methods and enhance the credence in its software components to generate appropriate results	[144]
2.	LigGrep	Tool for filtration of docked stances to enhance VS hit rates	Provides better hit rates in terms of test VS in targeting H. sapiens poly adenosine diphosphate ribose polymerase 1 (HsPARP1), *S. cerevisiae* hexokinase-2 protein (ScHxk2), and *H. sapiens* peptidyl prolyl cis trans isomerase NIMA-interacting 1 protein (HsPin1)	[145]
3.	LS-align	On an atomic level, flexible ligand structural alignment algorithm for high-throughput VS	Produces rapid and accurate atomic-level structural alignments of ligand for particular molecules	[146]
4.	ProPose	Navigated VS via Simultaneous Protein–Ligand Docking and Ligand–Ligand Alignment	A combined method based on ligand and receptor to ensure steric fit through VS via ranking the molecules as per their similar interaction pattern with known ligands	[147]
5.	StackCBPred	Stacking-based assumption of binding sites between carbohydrates and proteins from their sequence	Predicts biostructural features of amino acids to train a stacking-based ML effectively for the exact identification of binding sites between carbohydrates and protein s	[148]
6.	TrixX	Structural molecular indexing for large-scale VS in almost linear time	One of the fastest VS tools currently available, which is almost two times faster than standard FlexX	[149]

**Table 2:** List of MMs applied for COVID-19 management with their purposes.

No.	Model	Purpose	Reference(s)
1.	Bats–Hosts–Reservoir–People (BHRP)	Simulation of virus transmissibility from bat to human	[163,164,165,166]
2.	SPSS modeler	Predicting the number future infections, deaths, and tourism crises and disaster management (TCDM)	[167,168,169]
3.	Markov Chain Monte Carlo (MCMC)	Transmission dynamic model combined with personal protective measures	[170,171,172,173]
4.	Ordinary differential equations (ODE) metapopulation model	Disease transmissibility prediction and effect of dynamic interventions	[174,175]
5.	Susceptible–Exposed–Symptomatic–Asymptomatic–Recovered/removed (SEIAR)	Quantification of the age-specific ability for transmission and effect of personal protective measures	[176,177]
6.	Susceptible–Exposed–Infectious–Quarantined–Recovered (SEIQR)	Disease transmissibility prediction and management techniques	[178,179]
7.	Susceptible–Exposed–Infected–Removed (SEIR)	Prediction of disease transmissibility, epidemic scenario, and impact of humidity and temperature	[180,181,182,183,184,185]
8.	Susceptible–Infected–Recovered (SIR)	Monitor transmission and recovery rates in real time, as well as data fitting and management techniques.	[186,187]
9.	Susceptible–Infectious–Quarantined–Recovered (SIQR)	Strategies for management and measurement for quarantine	[188,189]

**Table 3:** List of MLs applied for COVID-19 management with their respective accuracies.

SN	Name of Tool/Developer/User	ML Algorithm	Accuracy (%)	Reference
1.	Deep Learning with X-ray	CNN	96.78	[190]
2.	iSARF	DT/RF	87.9	[191]
3.	Kunhua	LR	87	[192]
4.	NLR&RDW-SD	LDA	85.7%	[193]
5.	ResNet50	CNN	96.1–99.7	[194]
6.	COVNet	CNN	95	[195]
7.	COVID-Net	CNN	92.6	[196]

## Data Availability

Data will be made available upon request.
